# A novel multi-layer security framework for medical image transmission in cloud-based wireless body area networks (BAN) using XAI-guided obfuscation and WDTW encryption

**DOI:** 10.1038/s41598-026-66954-8

**Published:** 2026-08-28

**Authors:** Asmaa Rady, Usama A. Gad, AAbdelhady A. Ammar, El-Sayed Soliman A. Said

**Affiliations:** Department of Electrical Engineering, Faculty of Engineering, Al-Azhar University, Nasr City, 11884 Egypt

**Keywords:** Computational biology and bioinformatics, Engineering, Mathematics and computing

## Abstract

The existing frameworks for medical image encryption in the context of BANs have three major limitations: encrypting the whole image consumes enormous amount of processing time; failure to integrate explainable AI for identifying the importance of regions and prioritizing the same for permutation and XORing and inability of verifying the lossless integrity of the decryption process. To tackle these limitations, this paper introduces a novel scheme called weighted dynamic transform encryption (WDTW) for adaptive encryption of medical images. Firstly, a model based on convolutional neural network (CNN) is trained using the medical image data set and used for understanding image features. Once the image features are learned using the model, Grad-CAM heat maps are produced, which are used to generate region importance maps for selectively performing block-wise permutation and XOR operations. Further, to guarantee the lossless integrity of the process, a verification module is introduced based on weighted dynamic time warping. The experimental results indicate that our framework gives NPCR of 99.61%, Entropy of 7.99, and reduces processing latency by 35% when compared to the conventional full image AES encryption method. The encrypted outputs successfully passed all seven NIST SP 800-22 tests and also demonstrated excellent resistance to known plaintext attacks. The minimum entropy, maximum correlation, minimum NPCR and average UACI values were found to be 7.999956, 0.000997, 99.6036% and 33.4681% respectively.

## Introduction

**Fig. 1:**
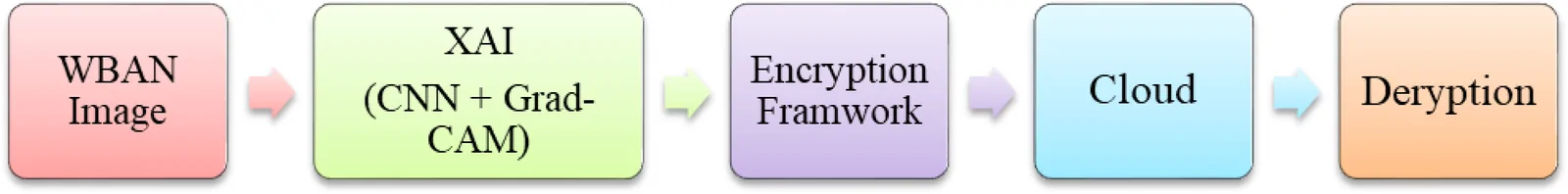
Architectural diagram

With the explosion in the usage of body area network (BAN) based devices in telemedicine practices, the vulnerability of private medical imaging data interception through wireless communication is on the rise^1^. While the encryption protocols such as AES^2^ are effective in providing layers of protection, the implementation of such standards within the BAN setup exhibits three basic flaws: (1) Uniform encryption: uniform encryption across the whole image will increase energy cost and processing delay, which will be counter-productive in terms of the resource limitation of the wearable sensors. (2) Absence of XAI Assistance: Without any way of identifying the importance of biomedical zones such as tumors or fracture sites, there will either be an inadequate level of encryption in the vital areas or overkill in the low importance zones. (3) Absence of Image Validation Process: Existing methods do not include any means by which the decryption process can validate whether the decrypted image is an exact copy of the original one. This work is solely focused on static medical images in BAN-edge computing environment. We present an adaptive medical image encryption architecture which is based on four major pillars as follows: First, Deep Learning using CNN^3^: A Deep Learning model through CNN algorithm is trained on a specially curated medical images dataset and helps in extracting deep features from medical images (tissues, edges, abnormalities). Second, Guiding using Grad-CAM^4^: Model’s weights and gradients help in creating heatmaps that identify the most critical regions used in the classification, which leads to identifying highly accurate critical regions which give real-world clinical insight into the image. Third, selective and adaptive encryption (WDTW): Heatmap created above is given as weighted masks to the new WDTW encryption algorithm that selectively permutates and performs XOR operations only on critical regions to reduce computation cost and secure vital regions. Fourth, lossless verification (DTW): We have developed a novel verification mechanism using weighted dynamic time warping (DTW) technique to compare the decrypted image with the original one and ensure perfect reconstruction. Medical data captured by WBAN devices vary significantly based on the type of sensor employed and the required clinical task. Electrical sensors are utilized to capture time-series biosignals such as electrocardiogram (ECG) and Electroencephalogram (EEG). In the context of internal anatomical imaging, ultrasound sensors produce cross-sectional images of soft tissues, whereas pill cameras provide endoscopic images of the gastrointestinal tract, and 3D sensors contribute to constructing comprehensive volumetric organ models. Since this research focuses on high-resolution static medical imaging applications, we specifically limit our study to Ultrasound and 3D images, as they are most suitable for implementing AI-guided encryption frameworks (e.g., Grad-CAM) that rely on spatial feature extraction, unlike temporal signals (e.g., ECG/EEG) which require fundamentally different processing and verification mechanisms (Fig. 1).

The proposed algorithm would employ the CNN prediction explanation obtained using Grad-CAM in order to find the class-discriminative sensitive areas of the image. This is because the technique provides localization maps showing the parts of the image that are responsible for the decision made by the CNN. The localization maps obtained will be converted to protection scores per block, and only semantically important blocks will be considered for WDTW permutation/encryption. This is different from selective image encryption methods where the area of an image requiring encryption is selected either manually or using some saliency criterion, since it selects the semantically important regions of the image.

In addition, the proposed research will combine the XAI-driven block selection mechanism along with the WDTW based block transformation and a reversible post-diffusion step. The implementation includes evaluation of entropy, correlation, NPCR, UACI, key sensitivity, NIST style randomness testing, and plaintext-resistance properties.

The proposed algorithm is implemented by a graphical user interface (GUI)—as a dashboard—developed by the Tkinter library in Python programming. This provides an interactive interface that allows users to interact with the program easily.

The rest of the paper is organized as follows: In “Literature review” section discusses a last recent related works. In “Problem definition” section formulates the research problem by defining the major challenges and gaps present in the existing approach. In “Research method” section describes the method used to analyses data, in “System implementation” section explains the deployment and development of the proposed algorithm, in “System analytical modeling” section provides an analysis of the system model using mathematical equations to explain its behavior and interactions among the components and in “Results and discussion” section describe the findings that have been obtained from the implementation.

## Literature review

The research^5^ aims to develop an integrated security system to protect medical data (especially medical images) during storage and transmission across networks, by combining three key technologies: Multi-source image fusion (MRI + SPECT/PET), Double watermarking and Data encryption. The research suggests three methods: first stage is Medical Image Merging, second stage Dual Watermarking in these stage two types of information are hidden in the merged image; there are Identity Watermark and Integrity Watermark using LWT, HD and SVD techniques and Chaotic Hennon-Sen Map.

The research^6^ presents a revolutionary and all-encompassing steganography architecture that makes use of the generative adversarial network deep learning algorithm, and the discrete cosine transform (DCT). The suggested hybrid architecture provides a reliable solution for applications needing high levels of security and integrity of data by utilizing machine learning techniques in both the spatial and frequency domains. Although traditional steganography techniques are often divided into spatial and transforming domains, a thorough investigation and analysis show that the hybrid approach performs better than separate techniques.

Chaudhary et al.^7^ propose a strong watermarking system that combines Arnold Scrambling, Hessenbergy decomposition (HMD), singular value decomposition (SVD), and discrete wavelet transform (DWT). The suggested approach guarantees little damage to picture quality by using DWT to break down the medical image into frequency sub-bands and putting the watermark into the most important sub-band. While SVD collects and modifies the image’s key elements, HMD simplifies the sub-band matrix. Before embedding, the watermark picture is further secured using Arnold scrambling. The suggested method is appropriate for protecting medical photographs in digital settings, as it strikes a compromise between robustness and imperceptibility. The suggested approach has been used on several medical datasets, and its robustness and imperceptibility have been assessed.

With the expansion of IOTs and Wireless devices^8^, data transmission has increased over the internet. Maintaining security during data transmission using invisible watermarks has become a focus and has captured the attention of numerous researchers. The three security necessities, privacy, integrity, and authenticity, call for close attention and remedial actions. In this respect, watermarking and cryptographic techniques are a probable way to handle the above-mentioned issues adequately. The paper presents a novel approach to watermarking using a combination of NSST, HD, and SVD transforms, which improves the robustness and security of the watermarked image. This technique makes use of a gray scale image as the watermark and embeds the same in different cover images. These images then go through the process of transformation by means of NSST, HD, and SVD to finally obtain the watermarked images. AES encryption is also performed by the authors to improve security and create a hash sub-key and an authentication tag. It can be seen that the proposed technique outperforms others in terms of efficiency by about 47.81% maximum.

For researchers aiming to ensure the safety of the data in the field of medicine from any form of tampering, the growing nature of such data is a major challenge^9^. The current research proposes a scheme based on dual image watermarking, which will enable hiding two different secret images in one host image at a time in order to solve this pressing problem in the domain of medicine. To enhance the embedding process, the proposed scheme utilizes a synergistic integration of deep learning network with 12 layers, NSST, and HOSVD. Experimental results have proven that the proposed framework outperforms existing schemes in imperceptibility, robustness, and high payload capacity.

The exponential growth of internet technology has brought about a revolutionary shift in the digital communication paradigm with images becoming one of the leading means of exchanging information on various online platforms^10^. This has made it essential for researchers to focus on protecting copyrights and ensuring the authenticity of digital images, but this is proving to be increasingly challenging. While watermarking techniques have been common in the past, they lack in their ability to deal with the new age attacks that use artificial intelligence to launch complex attacks that can damage or even bypass watermarks. Architectures of Convolutional Neural Networks like AlexNet have been successful in producing discriminative deep features capturing high level semantic representation of digital images. However, such algorithms continue to be susceptible to geometric transformations like rotation, scaling, and cropping that adversely affect the watermark integrity and detection ability. In contrast to the deep learning methods, transform domain techniques-especially those involving Polar Harmonic Transforms-have been studied widely in the past due to their rotation invariance property. The GPHT algorithm along with its more computationally efficient versions provides stable feature descriptors that are robust to geometric transformations. However, such transform-based algorithms do not provide rich semantic information required for robust copyright verification through geometric attacks to the signals. In order to combine the benefits of both worlds and overcome the drawbacks of each, there is increasing research focus on hybrid algorithms where deep learning algorithms are combined with transform domain descriptors. Additionally, the utilization of chaotic encryption techniques through tent map scrambling is one of the techniques being researched to increase security of the watermark system. Even after considering all these improvements, the current available techniques mainly focus on using either deep learning techniques alone or transform-based techniques alone. This means that there is a research gap concerning approaches which would consider the issue of robustness against attacks using AI, robustness against geometric transformations, and the problem of cryptography together. As a solution to this research gap, a hybrid zero-watermarking technique has been proposed where AlexNet-based deep learning features have been used along with Fast generic polar harmonic transform (FGPHT). Moreover, a chaotic tent map encryption has also been used along with the FGPHT. From the experiments conducted, it is found that the performance of this hybrid approach is better than the current approaches.

Security of medical records has become an important issue in the tele-health system^11^. Driven by the significance of such important issue, we have come up with a robust watermarking scheme in the DWT-SVD-optimization framework. To overcome the issue of security, the traditional watermarking schemes employ manual scaling factor to maintain a balance between imperceptibility, robustness and capacity. However, the employment of manual scaling factor results in the loss of optimum trade-off between the above stated parameters of watermarking. In our proposed scheme, the medical data protection is achieved through the concept of dual watermarking and optimization process. In our proposed scheme, the data owner uses dual watermarks which are embedded into the medical image imperceptibly for achieving additional security. In this scheme, some optimal schemes are used to determine the scaling factor for watermark embedding. In addition, the performance of the scheme is analyzed and compared. Additionally, text patient information is encoded using the hamming code prior to being embedded into the cover in order to prevent any bit error.

Wearable devices and medical systems in the e-healthcare environment allow the transmission of biological signals from healthcare professionals to patients, thus providing collaborative medical practice, telemedicine, and medical expertise sharing^12^. Medical records play an important role in the implementation of telemedicine tasks such as clinical consultation, training of health care practitioners, and medical research. Intelligent healthcare systems use modern information and communication technology to provide smooth transfer of confidential medical information through networks. The growing frequency of healthcare data breaches has become an important crime with considerable financial consequences. It is necessary to protect the integrity and the intellectual property rights of the medical images in order to avoid their illegal replication, modification, and distribution. In response to this problem, many cost-effective watermarks have been designed for biomedical signals. It also offers an extensive overview of principles of watermarking that includes basic properties, different applications, general procedure, evaluation measures, and biomedical signal classes. Moreover, the article offers a review of the available watermarking methods for biomedical signals, which have been categorized depending on the type of signals. In this review, the novel watermarking methods along with their trends are critically analyzed as far as new challenges of their practical application are concerned. All the presented methods have been summarized systematically in the form of tables that show different applications of watermarking using biomedical signals and performance measures of those methods.

The rapid adoption of IoT devices and consumer electronics in healthcare has improved medical diagnosis, teleconsultation, and the transfer of important patient information^13^. However, the maintenance of privacy and validity of private medical images such as MRIs and X-rays has been a challenging issue. We propose an innovative dual watermarking system through deep learning which provides both imperceptibility and robustness. The proposed design uses a normalizing flow based encoder to efficiently embed watermarks into cover photos, whereas the dual-decoding framework guarantees the successful extraction of the hidden image with minimum degradation. A secondary level of security is given to the encoded image and a noise layer is introduced between the encoder and decoder to increase resistance to attacks. Exhaustive experiments performed on Tiny ImageNet and HAM10000 databases prove the efficiency of our proposed algorithm. The proposed algorithm has a superior performance compared to other watermarking algorithms in terms of PSNR and SSIM metrics, obtaining PSNR and SSIM values of 77.56 dB and 1.0, respectively. Our comparative analysis demonstrates that the proposed algorithm has average cover loss and secret loss values of 9.56% and 8.67%, respectively, while having improved correlation values of 0.9894, which is 0.194% better than previous algorithms.

The emergence of new watermarking schemes for medical images using deep learning has received considerable attention among researchers owing to the fast-paced advancement in deep neural networks^14^. There is more need now than ever before to offer robust copyright protection of medical photos, especially chest X-ray radiographs. Nonetheless, a large number of existing techniques are unable to optimize the embedding and extraction process of the watermark by exploiting the intrinsic features of the image. In this paper, we propose a novel framework for dual watermarking based on deep learning with the help of the Redundant Discrete Wavelet Transform (RDWT). The proposed framework integrates a normalizing flow technique in the encoder component for an elegant watermarking process in a medical cover photo. An additional layer of security is introduced to enhance the protection of the image after encoding it. Resilience of the system is further enhanced by introducing a noise layer in between the encoder and decoder specifically to facilitate secure image extraction. The experimental results clearly show that the proposed method exhibits better watermark robustness and invisibility than current state-of-the-art techniques, with the loss rate of pixels being less than 0.1% in many cases. The research carried out on the NIH ChestX-ray database proves that the quality of the recovered secret images is visually similar to that of the original secret images. The comparative analysis once again proves the superiority of the proposed method.

It is important for the existing health care system to have protection and assurance through the use of data since there is widespread use of digital patient records and exchange of personal patient information^15^. It is very crucial to ensure that the privacy and security of the medical images are preserved since they have a very important part to play in diagnosing patients, creating treatment plans, and conducting research. But, the problem is that since these images are digital in nature, they can be easily accessed, tampered with, and misused. A resilient dual watermarking framework which employs both visible and invisible watermarks through the combination of Non-Subsampled Shearlet Transform (NSST), Bi-dimensional Empirical Mode Decomposition (BMEMD) and Multi-Scale Singular Value Decomposition (MSVD) has been developed. This algorithm implements Redundant Discrete Wavelet Transform (RDWT)-Randomized Singular Value Decomposition (RSVD) encryption scheme and incorporates visible ownership watermark along with invisible authentication watermark. The excellent resistance to various attacks such as additive Gaussian noise, JPEG compression, and blurring has been established through experiments conducted on a subset of 3000 images from the NIH Chest x-ray dataset. A PSNR value of 71.4742 dB and an SSIM value of 0.9997 establish the effectiveness of the proposed scheme in maintaining high visual quality while registering a gain of 47.49% in Normalized Correlation (NC) value. These findings prove the potential of the proposed method in ensuring the secure transfer and authentication of medical images.

Owing to the fact that ECG signals use digital transmission and have no inherent security, they can be manipulated, falsified, or accessed without permission^16^. Results from this study, current data deter deterioration, telemedicine, deterioration, telemedicine, deterioration, telemedicine, telemedicine, telemedicine, deterioration, telemedicine, telemedicine, deterioration, telemedicine, strong deterioration, telemedicine. The 1-D ECG is transformed to 2-D extracted features, redundant discrete wavelet transform, and multiresolution singular value decomposition through the Pan-Tompkins++. Watermarks are encrypted using the changed parts. Denoising Convolutional Neural Networks give protection against typical attacks but preserve signal integrity by denoising the extracted watermarks. The peak signal to noise ratio n is 83.48 dB and the normalized correlation value is 0.9991, which proves experimental improvement over other approaches in terms of better resistance to typical attacks such as Gaussian noise, Speckle noise, and JPEG compression.

The rapid development of communication technology along with POC services has promoted the exchange of digital biomedical documents via the open network for remote health care support^17^. Biomedical documents consist of private and secret information of patients. It is very difficult to keep the information away from any kind of unauthorized distribution and manipulation while it is being transmitted. This paper introduces the encryption based non-blind watermarking technique to provide security for biomedical signals. A robust and secure algorithm is developed for watermark embedding in ECG signal via contourlet transform and multi-resolution SVD. In the first stage, the mark image is coded through the application of the chaotic shift transform and modified henon map based encryption method to enhance the security of the proposed scheme. Afterwards, the encoded watermark is embedded into the higher frequency components of the ECG signal so as to avoid the ownership problem. In order to assess the performance of the proposed algorithm, the standard ECG MIT-BIH and PTB Diagnostic ECG databases are used. The performance of the proposed scheme is analyzed against the state-of-the-art literature, and the results have shown an improvement of up to 28.98%. It can be seen that the presented schemes deliver promising performance with regard to robustness, invisibility, and security.

Although digital images can be distributed and replicated easily, it may be difficult to prevent identity theft, privacy violation, and ownership problems^18^. Medical image encryption provides an essential function in securing the privacy of sensitive information and protecting the privacy of patients. The present study tries to tackle this problem by presenting an innovative solution for medical image security through the application of chaotic key fusion and secret-sharing method in order to provide security for medical images. One of the key points of this work is the utilization of Rubik’s cube-based bit-plane shuffling method in order to decrease the computational load of image encryption and add a new perspective to the field of medical image security. Another point that makes our work distinct from other works is the use of the segmentation process in order to increase efficiency and decrease the time complexity of the system. Proposed encrypted image sharing has been done through the K–N Secret sharing algorithm, which helps in providing authentication and robustness. The proposed decrypted image is further improved with super resolution to provide better information output. Proposed technique has been proven secure and provides better results along with being easy. The average values of NPCR and UACI for the proposed encryption technique are 99.47 and 49.90, respectively, and the entropy is 7.995, thus proving the robustness and effectiveness of our proposed approach. The proposed technique has been found to be highly sensitive towards the keys bit and average time complexity.

As a result of increasing the number of digital information technologies, the people have started to use the internet to convey their data^19^. There are many threats to this type of data through which attackers can steal this sensitive data of the people. The technique of encryption provides a security level to protect the data. In this research, the data under discussion will be of images. There are many information pixels stored in the image and correlation between them is high. Hence, in this research paper, an image encryption technique is proposed which will employ the Henon map along with osprey optimization (OO) algorithm. OO approach tuned the parameters optimally by analyzing the solution space for the parameter based on the entropy objective function and then choosing the optimal one. Moreover, in the proposed encryption approach, two random sequences are generated using the chaotic Henon mapping. Then, these sequences are used in order to implement the confusion and diffusion process in the proposed algorithm. Simulation results show that the histogram of the encrypted images is uniform distribution, entropy with high values close to the optimal value is needed in the encryption process, and low values of CC, SSIM, and PSNR are obtained from the proposed technique.

In^20^ the ECC method is combined with biometric encryption. A dual encryption technique is created by utilizing the uniqueness of biometrics and the benefits of the ECC algorithm for resource-constrained contexts. Biometric encryption is utilized as the initial processing, and ECC is used as the secondary encryption, which increases security. In addition, it offers strong scalability and compatibility, lowers resource usage, increases node life, and can be integrated with devices that already have architecture.

By supporting an innovative three-layer security system and carefully using the power of machine learning approaches^21^ tackles this urgent problem. To achieve cross-tier security, the device, hub, and cloud levels must be deliberately targeted. Innovative in addition to improving security at the device layer, the Sense Guard anomaly detection system strategically supports patient wellbeing and the WBAN network thanks to advanced machine learning algorithms. Concurrently, the Hub layer is strengthened by a novel intrusion detection method, and the Cloud layer’s adaptive machine learning models guarantee a thorough defense. Cross-tier security is at the forefront of WBAN innovation thanks to this strategy, which offers an effective solution to the security issues inherent in healthcare IoT as a significant achievement in improving the state-of-the-art.

The research^22^ considers the latest developments in artificial intelligence (AI), which present both new attack vectors and chances to improve cyber resistance. Additionally, the effects of certain regulatory actions, such the European AI Act, are considered. The idea of a ring topology is put forth, which concatenates data in the form of a “data train” and thus produces faster and more effective communication, in contrast to, for example, the suggested star network topologies of the IEEE 802.15.6 WBAN standard or the Technical Committee (TC) Smart BAN of the European Telecommunication Standards Institute (ETSI). Beyond that, the strategy for adding an additional channel considers the conductivity of human skin. In addition to strengthening the system’s security, this necessity for direct interaction makes it easier to communicate securely, which is essential for preserving the integrity of private health information. To optimize security, the effort discovers several threat models related to the WBAN system and assesses possible risks and data vulnerabilities. Using the token-based technique as a case study, it emphasizes the critical balance between efficiency and security in WBANs. Additionally, it lays the groundwork for next developments in healthcare technology with the goal of guaranteeing the safe and effective integration of patient data.

The work^23^ focuses on using Support Vector Machine (SVM)-based detection of anomalies to improve security in WBANs. The primary issue discussed is the lack of focus on security protocols in WBANs, especially regarding secure connections and mitigating techniques. To ensure continued use, the suggested solution uses SVM to classify security measures for WBAN telehealth systems based on pertinent features. The main findings demonstrate SVM’s efficacy in boosting security in WBANs by successfully predicting vital signs with an astounding accuracy of 98.63%. The use of support vector machines (SVMs) to improve WBAN security and intelligence updates is examined in this article. In times of crisis, some access management strategies could work better. This study ensures the continued use of security measures for WBAN telehealth systems by classifying them exclusively using SVM based on pertinent security criteria.

El-Adawi et al.^24^ proposed new system for recognizing human activities inside a body sensor network setup. They changed one dimensional readings from accelerometers gyroscopes and magnetometers into two dimensional images using the GAF approach and then ran those through a DenseNet model already trained on other image data. The experiments used recordings from ten people doing twelve different actions such as walking and cycling with sensors placed on the chest wrist and ankle. Their results hit 97.83% accuracy along with matching F measure and a slightly lower MCC score which beat the other models they checked against. It seems putting all the sensor inputs together gave much stronger performance than any single one and the magnetometer by itself still reached around 87%. This kind of image transformation seems to let the CNN models work better overall and the DenseNet choice probably helps avoid some training problems while letting features get reused more. I think the vanishing gradient part matters here but the WBAN side feels a bit less clear in how it connects. Some of the comparisons might depend on exactly how the data was split though.

Yousuf et al.^25^ looked at how much better advanced security steps work compared to basic ones when it comes to keeping cloud data safe. They pulled together results from quite a few papers covering breaches over several years. The basic steps like encryption at rest cut the chances by quite a bit while multi factor checks and fixing configurations also helped noticeably. Advanced options seemed to push those numbers higher overall. Zero trust setups stood out the most but the others like posture management tools and runtime checks for containers still made a clear difference. It feels like the gains show up stronger in certain areas such as finance or health when using infrastructure services. In software as a service setup though once the basics are in place the extra steps do not add as much. A few things keep getting in the way of using the better controls. Not enough people with the right skills comes up often and budgets are tight for many teams. Legacy systems that do not fit well also cause problems. They put together some kind of way to decide where to spend on security based on the type of cloud service and the industry. The idea of adding automatic fixes into the main rules came up too. I think this part gets a bit messy when trying to apply it across different places.

Odeh and Abu Al-Haija^26^ gave an extensive review of the technical aspects of the various medical image encryption approaches, with particular attention paid to possible issues. This study was aimed at securing medical images (MRIs, CTs, chest X-rays) in terms of their secure storage and transmission through telemedicine. In this regard, a systematic analysis of 66 articles out of 4514 studies according to the PRISMA methodology was performed. Three major groups of the analyzed approaches were identified: Edge Maps, Watermarking, and Adaptive Medical Image Encryption (AMIE), with a comparison in relation to the CIA security triad (confidentiality, integrity, availability). As a result, it was found that the majority of the examined methods have high entropy (close to 8) and low correlation coefficients (less than 0.01), which means a high level of protection against statistical attacks (Table 1).

**Table 1:**
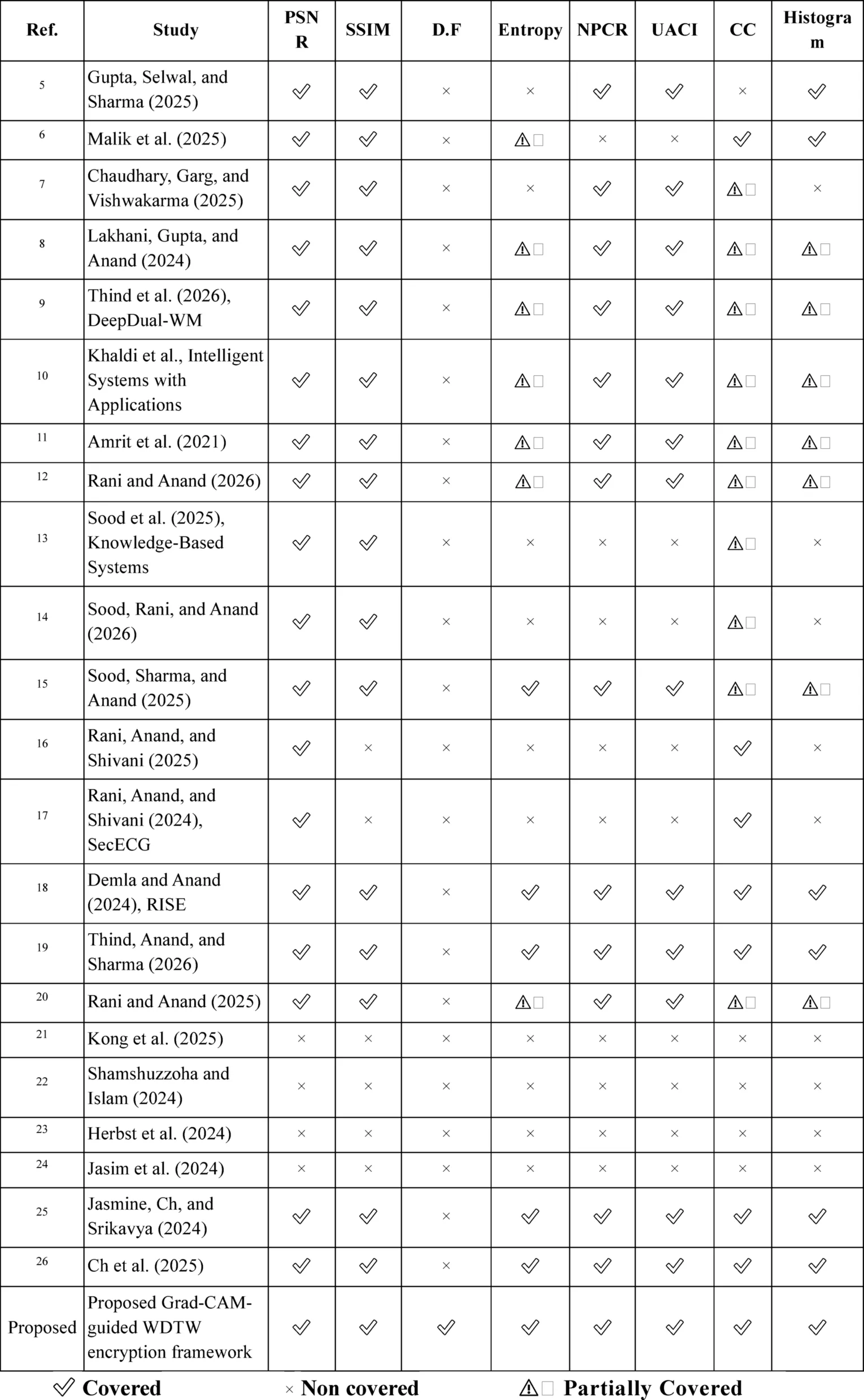
Literature review summary.

## Problem definition

Transmission of medical images over cloud platforms is associated with two major problems: one, security to maintain confidentiality; second, noise issues during transmission that affect the quality of images received. Any breach in security may lead to the breach of privacy of patients, and any noise present in the transmission of medical images may adversely affect their quality and lead to incorrect diagnosis. This study seeks to develop an effective model that will protect medical images from eliminate the cloud noise.

## Research method

**Fig. 2:**
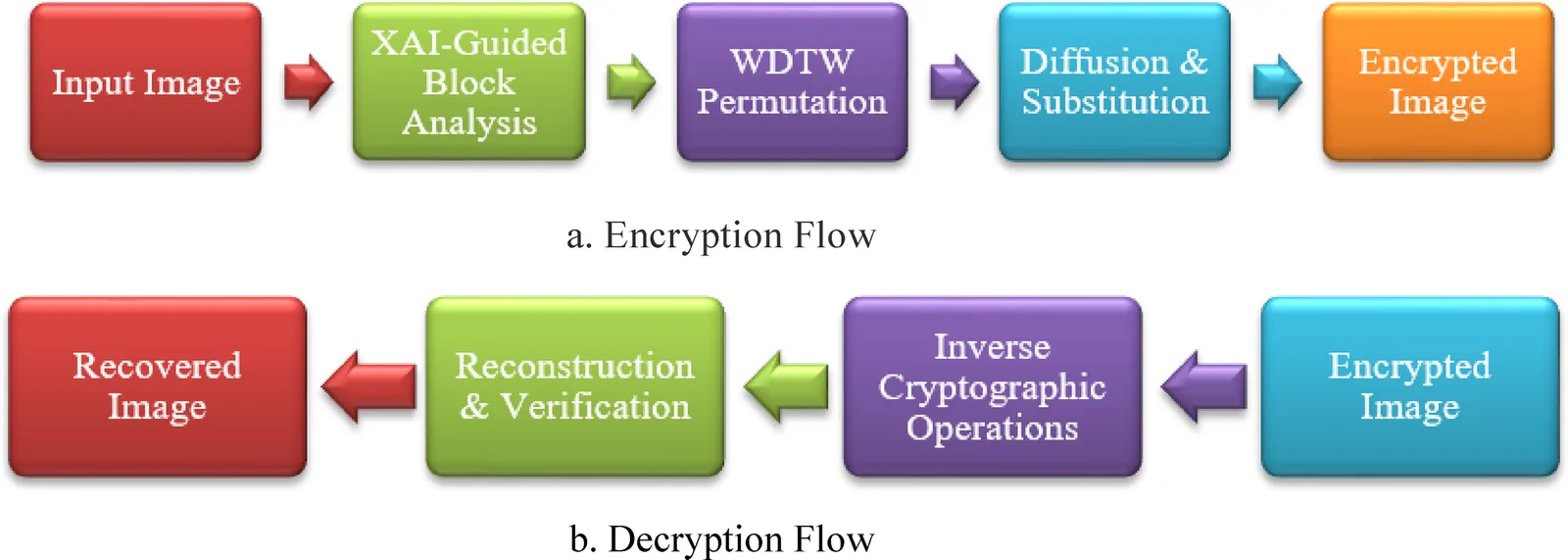
Logical workflow diagrams

The work of the proposed framework commences with the deep training phase, where a convolutional neural network (CNN) model is trained on a specialized medical image dataset. This training aims to enable the model to extract deep, discriminative spatial features from medical images, thereby making it capable of classifying them and understanding the visual patterns associated with various pathological conditions. Upon completion of the training phase, the trained model serves as the foundation for generating visual explanation maps. Specifically, when any new image is passed through the model, the Grad-CAM explainable visualization technique is applied to compute the gradients of the predicted class with respect to the feature maps in the final convolutional layer. This yields a numerical heatmap matrix that both visually and numerically highlights the region’s most influential in the classification decision, which in turn represent the diagnostically critical regions (e.g., tumors or fractures). To transform this spatial significance into an adaptive encryption mechanism, the system computes a pre-processed mask based on the grayscale version of the original image. This processing yields a numerical mask that represents weights assigned to different regions within the image. Finally, this mask is fed as an input to the WDTW encryption algorithm, where it is utilized to guide the block permutation process. The algorithm reorganizes the image units (blocks) according to the mask’s priorities, ensuring that blocks located in high-diagnostic-importance regions (with high heatmap values) undergo more complex permutation and scrambling operations, while less important blocks are subjected to lightweight or nearly negligible permutations. This ensures that the encryption burden is concentrated on the most sensitive regions, thereby reducing the overall computational overhead (Fig. 2).

The role of WDTW here is to create a source ordering that depends on the content of the blocks chosen by XAI. For each chosen block, a normalized structural sequence is derived based on the intensity values of the rows and columns in that block. The WDTW distance of this sequence and the sequence derived from the master key is then computed. The computed distances form the basis of the source ordering of the blocks. On the other hand, the destination ordering is created using the 256-bit master key independently of the XAI blocks. The domain-separated key material is generated for WDTW reference, the permutation of the destination, the substitution box, and the diffusion procedure. Therefore, the key space is 2^256^. The explanation is that the number of possible permutations of the blocks and substitution boxes cannot be multiplied by 2^256^ because all of them are deterministically generated using the same master key.

After the permutation following WDTW, the whole image is encrypted using key-dependent XOR masking and the substitution box. Two diffusion rounds are then performed in both directions. It adds the inter-byte dependence to the cipher text, and it improves the propagation of changes in the plaintext. The integrity is checked separately with HMAC-SHA256; WDTW similarity is used only as an additional structuring feature. Let P be equal to $P = 3H^{\prime}W^{\prime}$ and represent the number of image bytes, m the number of selected blocks, b the size of a block, L the length of the structural-sequence and w the WDTW window. the pixel-wise substitution and diffusion processes will still be linear with respect to image size but the main non-linearity will be due to sorting of selected WDTW scores.

## Techniques applied

### WDTW encryption mathematical method

#### Master-key generation

A 256-bit master key is sampled uniformly. HKDF-SHA256 derives purpose-specific key material. In Eq. (2), PRK is obtained as HMAC-SHA256 (salt, K), while $info_{x}$ provides domain separation^27,28^.

1 $$ K\mathop \leftarrow \limits^{{{\$ }}} \{ 0,1\}^{256} $$

2 $$ K_{x} = {\mathrm{HKDF}} - {\mathrm{Expand}}\left( {PRK,info_{x} ,L_{x} } \right) $$

where $K_{x}$ represent component-specific derived key material.

#### WDTW formulation

The weight is applied as in the WDTW formulation^29^.

3 $$ w_{{\left| {i - j} \right|}} = \frac{1}{{1 + \exp \left[ { - 0.25\left( {\left| {i - j} \right| - \frac{L}{2}} \right)} \right]}} $$

where I and j are sequence-position indices. The dynamic-programming search is restricted to $\left| {i - j} \right| \le r$ using a Sakoe–Chiba-type window^30^. and r is the window radius.

4 $$ c_{q} \left( {i,j} \right) = \left| {w_{{\left| {i - j} \right|}} \left[ {x_{q} \left( i \right) - r_{K} \left( j \right)} \right]} \right|^{2} $$

where $c_{q} \left( {i,j} \right)$ Local WDTW coast and, ${\mathrm{x}}_{{\mathrm{q}}}$ is a block feature sequence and ${\mathrm{r}}_{{\mathrm{K}}}$ the key-derived reference sequence.

Cumulative distance $\gamma_{q} \left( {i,j} \right)$ describe by

5 $$ \gamma_{q} \left( {i,j} \right) = c_{q} \left( {i,j} \right) + \min \left\{ {\gamma_{q} \left( {i - 1,j - 1} \right),\,\gamma_{q} \left( {i - 1,j} \right),\,\gamma_{q} \left( {i,j - 1} \right)} \right\} $$

Final WDTW score of block q $d_{q}^{WDTW}$ described by:

6 $$ d_{q}^{WDTW} = \sqrt {\gamma_{q} \left( {L,L} \right)} $$

7 $$ \pi \left( {\sigma_{i} } \right) = \delta_{i} ,\quad i = 1,2, \ldots ,s $$

where *π* Block permutation mapping, σ represent the source order, δ is the destination order of the key-driven Fisher–Durstenfeld shuffle^31^. Since both orders contain every selected block exactly once, the mapping is bijective and its inverse maps $\delta_{i}$ back to $\sigma_{i}$.

8 $$ \left| {\mathcal{K}} \right| = 2^{256} $$

Effective permutation entropy bound $H_{perm}^{effective}$ described by

9 $$ H_{perm}^{effective} \le {\mathrm{min}}\left( {{\mathrm{log}}_{2} \left( {s!} \right),256} \right) $$

10 $$ V_{i} = S_{K} \left( {P_{i}^{\pi } \oplus Z_{i} } \right) $$

where $P_{i}^{\pi }$ is the byte after block permutation, $Z_{i}$ the key-derived chaotic byte, and $V_{i}$ the byte after the first keyed substitution. The latest implementation then applies non-linear forward and backward diffusion using modular addition and bijective S-boxes.

Forward boundary state $F_{ - 1}$ described by

11 $$ F_{ - 1} = IV_{F} $$

12 $$ F_{i} = S_{F} \left( {\left[ {V_{i} + KS_{i}^{\left( F \right)} + F_{i - 1} } \right]\bmod 256} \right) $$

Backward boundary state $$C_{N} $$ described by

13 $$ C_{N} = IV_{B} $$

14 $$ C_{i} = S_{B} \left( {\left[ {F_{i} + KS_{i}^{\left( B \right)} + C_{i + 1} } \right]\bmod 256} \right) $$

#### Time calculation

Encryption time complexity

15 $$ T_{enc} = O\left( {N^{\prime} + sb^{2} + sLr + s\log s} \right) $$

Decryption time complexity

16 $$ T_{dec} = O\left( {N^{\prime} + sb^{2} } \right) = O\left( {N^{\prime}} \right) $$

where N′ is the total number of padded image bytes, s the number of selected blocks, b the block dimension, L the feature length, and r is the window radius.

The proposed algorithm includes a dedicated statistical and computational evaluation in which the proposed Grad-CAM-guided method is assessed under the same images, cryptographic parameters, block size, master-key policy, and hardware environment used for the random-block and intensity-based ROI baselines. Because the three selection strategies are evaluated on the same test images, the measurements are treated as paired observations. An overall Friedman test is applied at α = 0.05, followed, when significant, by pairwise Wilcoxon signed-rank tests with Holm correction. The results are reported using mean ± standard deviation, median and interquartile range, 95% confidence intervals, and appropriate effect sizes. Computational performance is measured by separating the principal stages of the proposed pipeline: Grad-CAM generation, block selection, WDTW scoring, the remaining encryption core, and decryption. Model loading, file I/O, GUI updates, image display, and post-processing security metrics are excluded from the latency measurements. Five warm-up executions are discarded and each reported configuration is measured over 30 independent runs using a high-resolution monotonic timer. Peak CPU memory, and GPU memory when applicable, are measured separately. Scalability is evaluated across multiple image resolutions and selected-block coverage levels, while throughput is reported in MB/s. This evaluation distinguishes the cost of XAI guidance from the cryptographic operations.

For measured run r, each stage latency is calculated from the difference between the high-resolution ending and starting timestamps of that stage:

17 $$ T_{i}^{\left( r \right)} = t_{i,end}^{\left( r \right)} - t_{i,start}^{\left( r \right)} $$

18 $$ T_{Grad - CAM}^{\left( r \right)} = t_{Grad - CAM,end}^{\left( r \right)} - t_{Grad - CAM,start}^{\left( r \right)} $$

19 $$ T_{Block Selection}^{\left( r \right)} = t_{Block Selection,end}^{\left( r \right)} - t_{Block Selection,start}^{\left( r \right)} $$

20 $$ T_{WDTW}^{\left( r \right)} = t_{WDTW,end}^{\left( r \right)} - t_{WDTW,start}^{\left( r \right)} $$

21 $$ T_{Encryption}^{\left( r \right)} = t_{Encryption,end}^{\left( r \right)} - t_{Encryption,start}^{\left( r \right)} $$

22 $$ T_{Decryption}^{\left( r \right)} = t_{Decryption,end}^{\left( r \right)} - t_{Decryption,start}^{\left( r \right)} $$

where ${\mathrm{T}}_{{{\mathrm{Grad}} - {\mathrm{CAM}}}}$ represents Grad-CAM heat-map generation, $T_{Block Selection}$ represents Block scoring and selection, $T_{WDTW}$ represents feature extraction and WDTW scoring, $T_{{{\mathrm{Encryption}}}}$ is encryption time, and $T_{{D{\mathrm{ecryption}}}}$ represents decryption time.

To avoid double counting, $T_{{{\mathrm{Encryption}}}}$ is defined as the cryptographic core that follows WDTW. The complete Grad-CAM-guided encryption latency is therefore:

23 $$ T_{Enc,e2e} = T_{Grad - CAM} + T_{Block Selection} + T_{WDTW} + T_{Encryption} $$

If HMAC and structural verification are included after image recovery, the complete decryption-and-verification latency is:

24 $$ T_{Dec,e2e} = T_{Decryption} + T_{Verify} $$

25 $$ T_{enc} = O\left( {N + slogs} \right) $$

26 $$ T_{dec} = O \left( N \right) $$

where $T_{enc}$: Encryption execution time or computational cost, $T_{dec}$: Decryption execution time or computational cost, O(⋅): Big-O notation, which describes how the computational cost grows as the input size increases, N: Total image size, usually represented by the number of pixels or image bytes, s: Number of selected image blocks, *łog s*: Logarithmic cost associated with operations such as sorting or permuting the selected blocks, and s *łog s*: Computational cost of sorting or arranging the selected blocks.

In the proposed technique the WBAN sensors send its data to the Access point, the access point sends the data to the medical center via cloud using Wi-Fi technology.

**Fig. 3:**
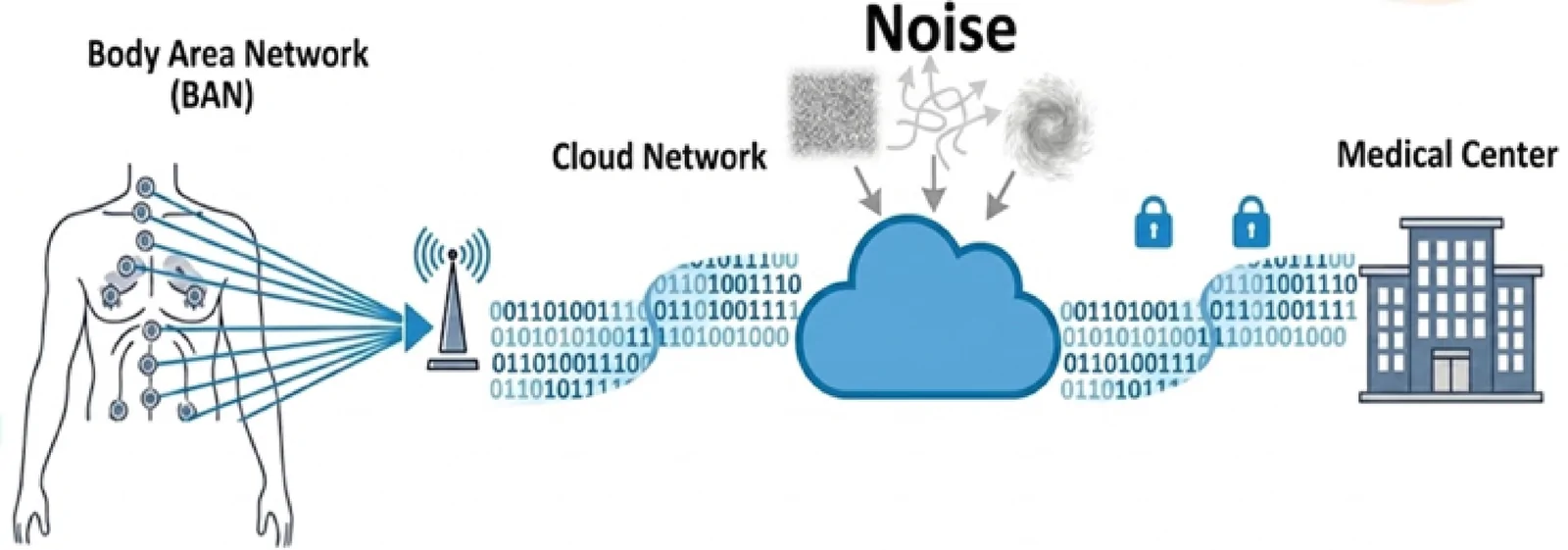
System model of the proposed scheme

The method proposed is to prevent the cloud data intrusion using two-layer of security:

1. Noise addition: the system considers two different types of noise; the user defined noise (UDN) that added by user after analyzing the image using XAI Grad-CAM technique.
2. Encrypt the image using WDTW technique, as shown in Fig. 3.

First layer encryption is adding noise to the sending image before encryption, and the second layer is encrypting the noisy image. XAI technique identifies medically sensitive or critical regions (such as tumors) to avoid modifying or encoding these areas in order to preserve the diagnostic value “*using Grad-CAM*”^32^. WDTW uses to ensure the compatibility with the nature of medical images. It will be dividing the sending image into blocks then applied a combination of encryption, permutation, and alpha blending at the block level. Finally, this approach distributes the processing across localized regions while preserving the structural integrity of diagnostically critical areas. In the proposed technique, noise is prevented within vital image segments where tumors and diagnostic regions are located. By ensuring that the sensitive regions are not disturbed by noise, this technique guarantees that the image will not be contaminated with any noise in the network.

In order to increase the stability of the suggested cloud framework against statistical attacks, and to ensure the integrity of data, a special noise method known as UDN has been developed. The noise mechanism is based on the combination of two statistical elements with different weights, which operate on the normalized image. First, a Gaussian component is generated using a random number generator with a fixed seed to ensure reproducibility. This component follows a normal distribution with zero mean and variance, mathematically expressed as:

27 $$ {\mathrm{G}}\sim {\mathrm{N}}\left( {0,\sigma^{2} } \right) $$

where G is the Gaussian noise matrix and σ is the standard deviation that regulates the strength of the noise. Second, a uniform noise component is added with the same dimension as the image. following a uniform distribution over the interval [− σ, + σ], given by:

28 $$ {\mathrm{U}}\sim {\mathcal{U}} \left( { - \sigma , \sigma } \right) $$

This element provides noisy elements that contrast sharply with the smoother elements in the Gaussian element. Finally, the two elements combine using a linear combination process, whereby the noise matrix *η* is formed from the equation:

29 * η = 0.6 G + 0.4 U *

The values 0.6 for the Gaussian noise and 0.4 for the uniform noise were chosen empirically in order to incorporate both natural and harsh noise features and, thus, make the statistical pattern more complicated. Fourth, noise matrix n is summed with the original image I to produce the noisy image $I_{noisy}$ by applying a clipping function:

30 $$ I_{noisy} \left( {x,y} \right) = clip\left( {I \left( {x,y} \right) + \eta \left( {x,y} \right), 0, 1} \right) $$

Concerning the regulation of noise intensity, a user interface has been provided for specifying a percentage P ∈ [0,100] such that it is mapped to the actual value of the standard deviation σ using:

31 $$ \sigma = \frac{P}{100} \times \sigma_{max} $$

Contribution to security: In terms of security, the use of such hybrid noise leads to the increase in the overall entropy value of the image and lowering of the correlation coefficient value of the spatial correlation between neighboring pixels. Thus, it is not possible for the intruder to guess the original structure of the data or perform any successful attacks based on statistics. This is supported by the results shown in the section with the calculation of entropy and correlation coefficients.

**Fig. 4:**
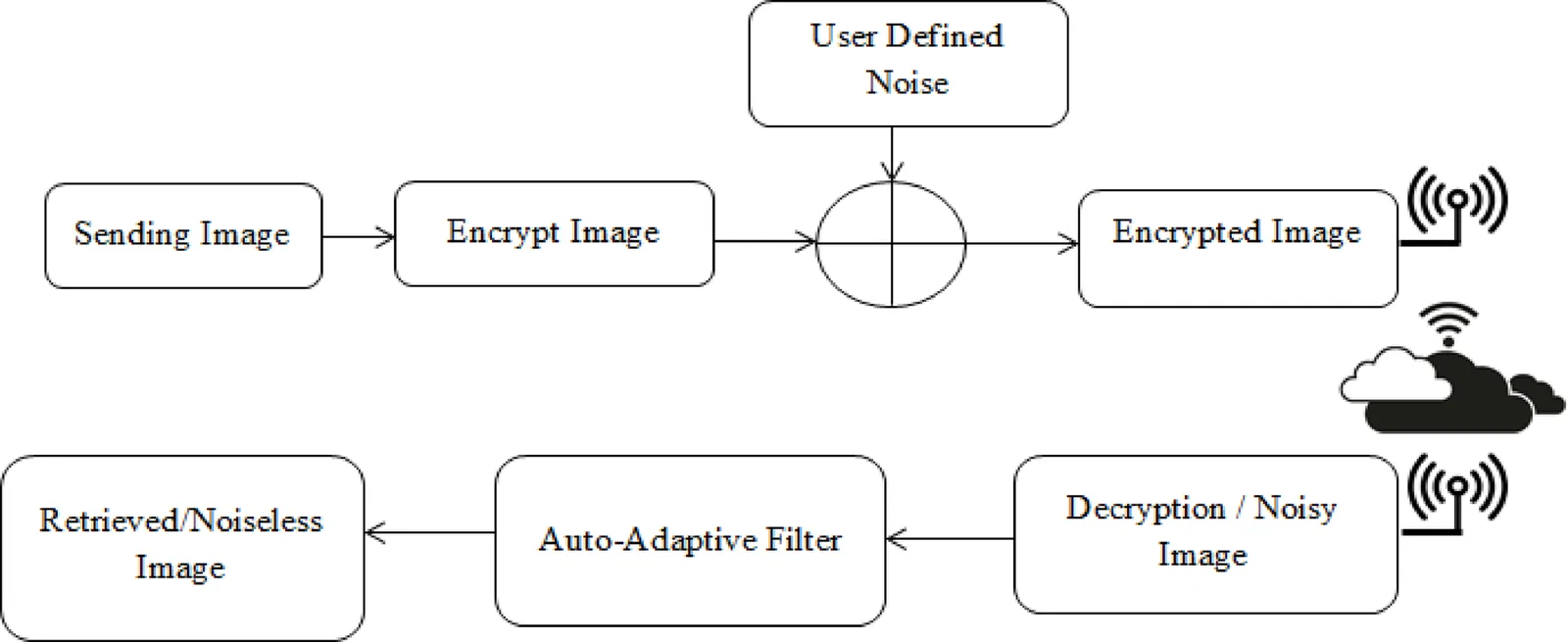
Block diagram of the model

In this model assume traditional noise (Gaussian noise, spackle, salt and pepper (S&P) and median) and security noise (Distributed Denial of Service DDOS, Side_channel, Crypto, Replication, Container, vm). The noise was added to the encrypted image. These noise types are intended to simulate some of the expected attacks on the image; the aim is to evaluate the system’s robustness under adversarial conditions. Finally, retrieve the sending image using suitable protection filters (Fig. 4).

### System implementations

The proposed system is mathematically modeled and software implemented using python, 16 GB RAM and processor core i5 CPU @ 2.5 GHz. Furthermore, full descriptions of the system using are procedures and pseudo code presented in the following paragraphs to demonstrate the main steps of the algorithm.

### Procedures

1. Image selection to be loaded, and then the image will be shown at the proposed dashboard.
2. XAI algorithm feeds the image to convolutional neural network model (CNN) to extract the features of image like; edges, textures, regions, then classify the image (for example: The medical image is Normal or has tumor) and detect the importance region using Grad-CAM technique, the Grad-CAM generate heatmap to determine the critical regions.
3. A pre-request security layer is UDN will be added to the image as an extra security level.
4. The WDTW assign four different block sizes (8 × 8, 16 × 16, 32 × 32, and 64 × 64).
5. The attacker noise or cloud noise will add through the channel.
6. Then the image will be decrypted and applied to the filter. Finally, system shows the Grad-CAM analysis and measures the metrics of retrieved image as in the dashboard.

**Table:** 

Algorithm 1: Pseudo Code of Secure Cloud Image Processing using Hybrid WDTW-XAI Algorithm	
**1. Initial image** → Padding and partitioning into blocks → CNN inference and computation of Grad-CAM → Extraction of important blocks → Feature extraction and calculation of WDTW score → Key-based permutation of blocks → Whole-image application of Chaotic XOR → Whole-image S-box operation → Post-diffusion of the image in both directions → Cipher image and integrity information **2. Cipher image** → Reverse post-diffusion → Inverse S-box operation → Inverse Chaotic XOR → Inverse block permutation → Reconstruction and cropping of image → HMAC validation and matching	

The CNN was implemented based on an ImageNet-pretrained MobileNetV2 backbone without its original classification head. RGB images were resized to 224 × 224 × 3 and rescaled to [− 1,1]. The backbone produced a 7 × 7 × 1280 feature map that was turned into a 1280-dimensional vector by global average pooling, batch normalization, dropout (0.25), 256-neuron ReLU layer with ${L}_{2}$ regularization (${10}^{-4}$), dropout (0.20), and a C-class softmax classifier. The last feature map of the CNN was used for computing Grad-CAMs. Direct class-folder datasets were partitioned with a stratified 80:20 train–test ratio and seed 42, whereas predefined dataset splits were kept unchanged; if possible, a stratified validation set of 15% of the training set was extracted. First, training was carried out with the backbone frozen using Adam at 3 × ${10}^{-4}$, then, whenever enough examples were present, the final 30 non-Batch-Normalization layers were fine-tuned using Adam at ${10}^{-5}$. Class weighting, modest data augmentation, early stopping, and learning rate decay were used. The network has 2,591,040 + 257C parameters, where 330,496 + 257C are learnable with frozen backbone; for binary classification tasks, these numbers become 2,591,554 and 331,010 parameters, respectively.

The Grad-CAM guidance was checked against two methods that covered the same areas: picking blocks at random and the usual way of choosing areas based on how bright they are. The Grad-CAM heatmap was turned into scores for each block. Blocks that scored higher than a certain level were chosen, long as they covered at least the minimum required area. For each picture the other two methods had to choose the number of blocks as Grad-CAM. All the settings for the cryptography were kept the same for all three methods. When the pictures had notes about the areas of interest the test used something called Dice. Iou to see how well Grad-CAM worked. If the pictures did not have these notes the test used a method to see how confident it was. The test also looked at things like entropy. How well the blocks were connected. It looked at the time it took to run the test. The results for all the pictures are shown as the average plus or minus how much they varied. This test checks if Grad-CAM is really better at finding the parts of the pictures rather than just making the whole picture look good.

### System analytical modeling

*Power signal to noise ratio (PSNR)* is ratio between the between the level of a desired signal to level of noise. The ideal value for PSNR is infinity. So, the higher value of PSNR means the higher quality^33^.

32 $$ PSNR = 10 \, log_{10} \left( {\frac{{MAX_{1}^{2} }}{MES}} \right) $$

where $$MAX_{1} $$ the maximum possible value of a pixel and MES is is the mean squared error.

Where* MSE “mean squared error”* is a statistics metric used to measure the difference error between the sending images and retrieved images. The ideal value for MES is zero. So, the lower value of MES means the higher quality.

33 $$ MES = \left[ {\left( {\frac{1}{h \cdot w}} \right.* \sum \sum \left[ {I\left( {i,j} \right) - k\left( {i,j} \right)} \right] ^{2} } \right] $$

where h, w are the dimensions of image (width, height). $I\left( {i,j} \right)$ is the value of pixels in sending image. $K\left( {i,j} \right)$ is the value of pixels in retrieved image. $\sum \sum i$ s the sum over all pixels^34^.

*Structural similarity index (SSIM)* is a metric that measures the similarity between the retrieved image and the sending image. SSIM values range from 0 to 1. So, the best quality achieved when the value of SSIM closely to 1^35^.

34 $$ SSIM\left( {x,y} \right) = \frac{{\left( {\left( {{2}\mu_{x} \mu_{y} + { }C_{1} } \right)*\left( {2\sigma_{xy} + C_{2} } \right)} \right)}}{{\left( {\left( {\mu_{x}^{2} + \mu_{y}^{2} + { }C_{1} } \right)*\left( {\sigma_{x}^{2} + \sigma_{y}^{2} + C_{2} } \right)} \right)}} $$

where *x * the sending image, y is the retrieved image, $\mu_{x} , \mu_{y}$ are the average pixel intensity in the window (0.8 for x and 0.75 for y), $\sigma_{x}^{2} , \sigma_{y}^{2}$ are the variance of the window in each image, $\sigma_{xy}$ is the covariance between the two window, $C_{1} , C_{2}$ are the Constant for numerical stability, to avoid division by zero. $C_{1} = \left( {0.01 *L} \right)^{2} ,C_{2 } = \left( {0.03 *L} \right)^{2}$ and L is the pixel range.

*Deformation factor* is a metric used to quantify the structural distortion or alteration between an sending dataset and its processed or transformed version (e.g., after encryption, compression, or filtering). The deformation factor values from 0 to 1. The ideal is zero. It is often derived from the Structural Similarity Index (SSIM) using the formula^36^:

35 $$ {{Deformation\; Factor}} = 1 - {{SSIM}} $$

*Entropy* is a statistical method for measuring randomness or uncertainty in a system. With regard to image processing and cryptography, Shannon entropy is used for calculating the distribution of pixels in an image.

36 $$ {\mathrm{H}}\left( {\mathrm{S}} \right) = \mathop \sum \limits_{i = 0}^{{2^{L} - 1}} p\left( {S_{i} } \right) \cdot log_{2} \left( {\frac{1}{{p \left( {S_{i} } \right)}}} \right) $$

where H(S) is the *Entropy* value, L represent the number of bits per pixel, $S_{i}$ is the pixel value at index I and *p* $\left( {S_{i} } \right)$ is the probability of occurrence (frequency) of pixel value $S_{i}$.

*Number of pixel change rate (NPCR)* is used to calculate the proportion of changed pixels in an image compared to the original image. It tells us the difference of the encrypted image from the original image on a pixel-by-pixel basis. A high NPCR (near to 99.6%) is ideal because it shows that most pixels have been modified.

37 $$ {\mathrm{NPCR}} = \frac{{\mathop \sum \nolimits_{i = 1}^{M} \mathop \sum \nolimits_{j = 1}^{N} D\left( {i,j} \right)}}{M \times N} \times 100\% $$

where M and N represent the image dimension, D (*i*,*j*) is the binary difference function.

*Unified average changing intensity (UACI)* The metric of UACI computes the mean level of difference between the two images; that is, it measures the change in the pixel intensity, irrespective of any changes in the positions of the pixels. An efficient algorithm will produce a result close to 33.33% UACI for an 8-bit image.

38 $$ {\mathrm{UACI}} = \frac{1}{M \times N} \mathop \sum \limits_{i = 1}^{M} \mathop \sum \limits_{j = 1}^{N} \frac{{\left| {C_{1} \left( {i,j} \right) - C_{2} \left( {i,j} \right)} \right|}}{{2^{L} - 1}} \times 100\% $$

M and N are image dimensions, C_1_ $$\left( {i,j} \right)$$ represent Pixel value of the original image at position (i,j), C_2_ $$\left( {i,j} \right) $$ represent Pixel value of the encrypted image at position (i,j) and $$2^{L} - 1 $$ Maximum possible pixel value.

*Scalability*

39 $$ T_{Block \, Selection} = O\left( {N^{\prime} + B} \right) $$

40 $$ T_{WDTW} = O\left( {SL\left( {2r + 1} \right)} \right) \approx O\left( {SLr} \right) $$

41 $$ T_{Encryption} = O\left( {N^{\prime} + S \, log\, S + Sb^{2} } \right) $$

42 $$ T_{Decryption} = O\left( {N^{\prime} + Sb^{2} } \right) \approx O\left( {N^{\prime} } \right) $$

43 $$ M_{total} = O\left( {N^{\prime} + B + SL} \right) \approx O\left( {N^{\prime} } \right) $$

where $N^{\prime}$ = number of padded image bytes, S = number of selected blocks, L = WDTW feature-sequence length, r = WDTW warping-window radius.

*Throughput* The computational efficiency of the proposed research was evaluated using throughput and peak-memory measurements. Throughput quantifies the amount of uncompressed image data processed per second and was calculated from the actual NumPy array size rather than the compressed PNG or JPEG file size. This distinction is important because compressed file size depends on image content and compression settings, whereas the array size represents the amount of data handled by the encryption pipeline. Two throughput values were considered: the end-to-end encryption throughput, which includes Grad-CAM generation, block selection, WDTW scoring, and the cryptographic operations, and the decryption throughput, which covers the inverse cryptographic operations required to recover the original image.

44 $$ Q_{enc} = \frac{nbytes\left( I \right)}{{2^{20} \cdot T_{Enc,e2e - latency} }} MiB/s $$

45 $$ Q_{dec} = \frac{nbytes\left( C \right)}{{2^{20} \cdot T_{Dec - latency} }} MiB/s $$

In Eqs. (1) and (2), $Q_{enc}$ and $Q_{dec}$ denote the encryption and decryption throughput, respectively; $nbytes\left( I \right)$ is the memory size of the original image array; *nbytes(C)* is the memory size of the encrypted image array; ${T}_{Enc,e2e-latency}$ is the total guided-encryption latency; and $T_{Dec - latency}$ is the measured decryption latency. A larger throughput value indicates that the framework processes more image data per second.

**Fig. 5:**
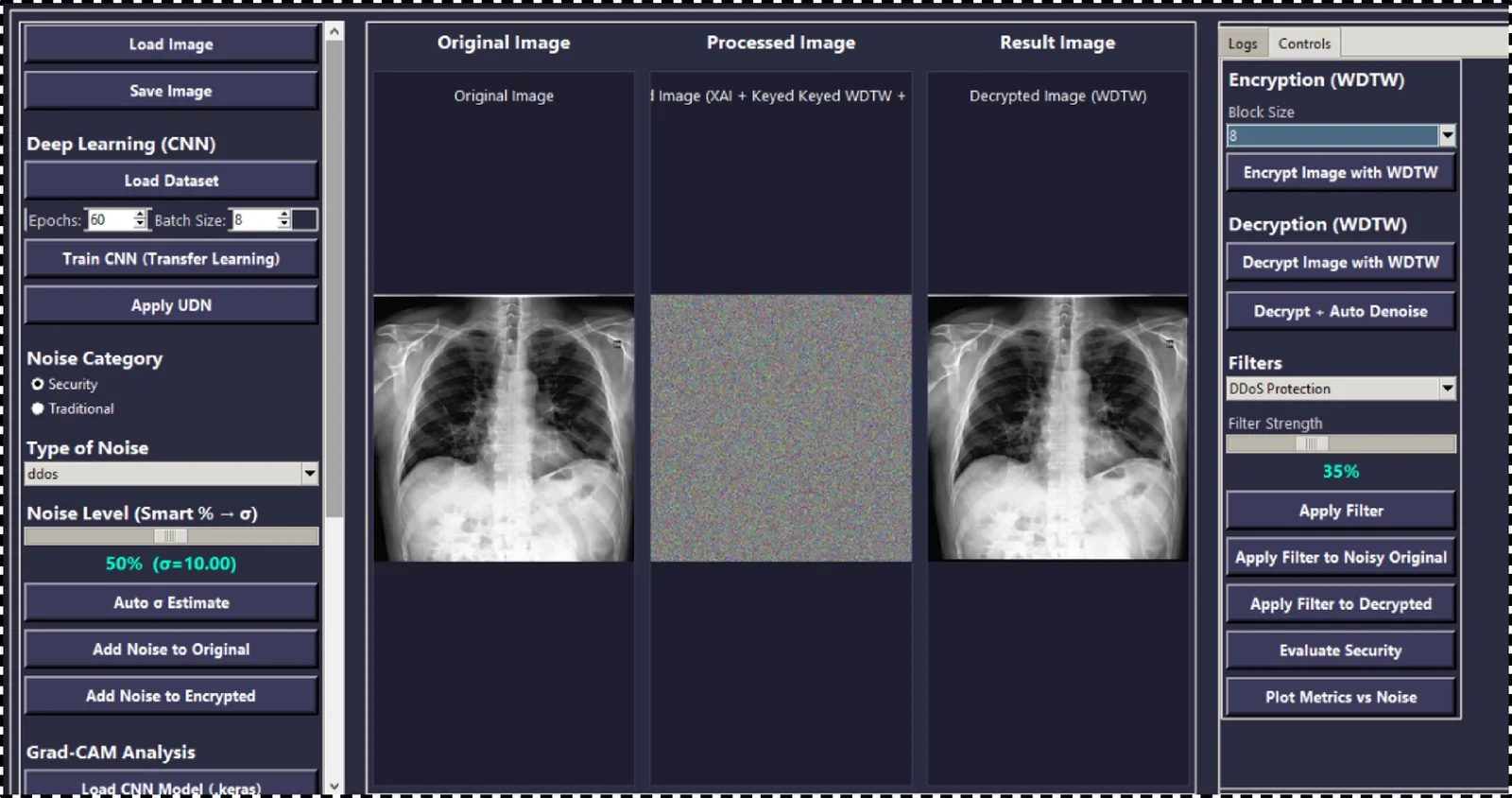
Graphical user interface (GUI) design of the system dashboard

*Memory usage* Maximum memory consumption was determined as the maximum increment in the resident-set size (RSS) of the process relative to the baseline taken right before the stage of interest. RSS is the amount of physical memory currently consumed by the process and includes the memory consumed by both Python and other libraries like NumPy, OpenCV, and TensorFlow.

46 $$ M_{peak} = maxRSS_{\left( t \right)} - RSS_{baseline} $$

where $$M_{peak}$$ denotes the additional peak memory required by the evaluated operation, $$RSS_{\left( t \right)}$$ is the process memory measured at time t, and $$RSS_{baseline} $$ is the memory usage immediately before execution. This measurement reflects the temporary memory required for the original and encrypted images, the Grad-CAM heat map, block-selection masks and scores, WDTW feature sequences, chaotic sequences, S-box operations, diffusion arrays, and the recovered image. Reporting the memory increase above the baseline is more informative than reporting the absolute process memory because it isolates the additional memory introduced by the proposed pipeline (Fig. 5).

GUI acts as an interface between the end user and the data under investigation. This system is fast response, lower memory size and easy to use. The GUI provides users facilities by the following:

- select the type of noise,
- adjust level of noise,
- applying encryption and decryption processes,
- select the suitable filter to apply,
- show grad-CAM analysis,
- Show the result metrics to measure the efficiency of system and show the image in all cases (Original, Encrypted, Decrypted/filtered).

## Result and discussion

The WDTW algorithm adapts block permutation techniques and encrypts images using high deformation factors with low SSIM values. The system was stress tested using a threat model representative of attack simulations. The system is therefore considered a highly protected were data was healthy.

**Fig. 6:**
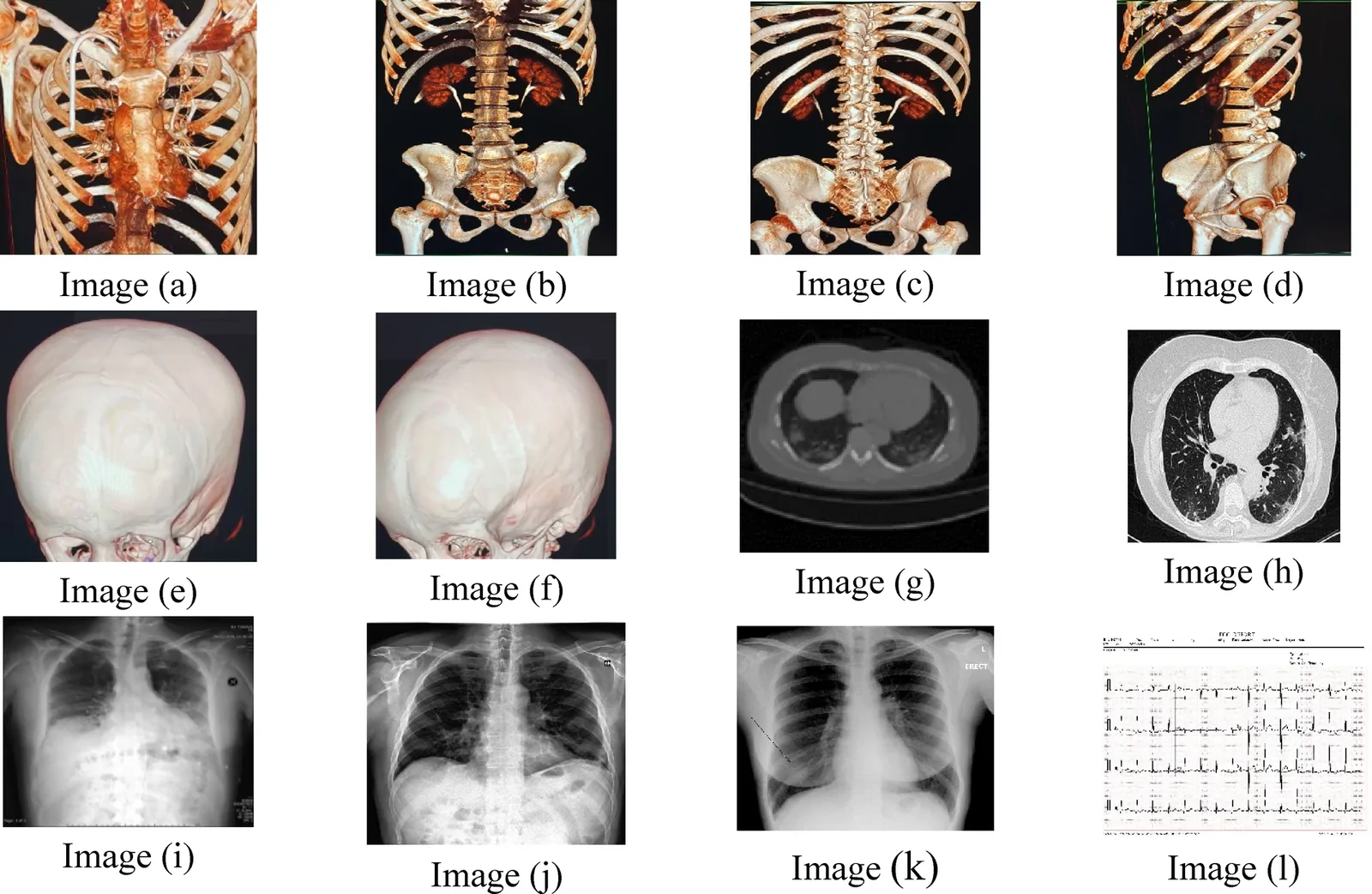
Sample of images taken as input

Figure 6, show samples of images that are considered the system dataset inputs to test the proposed system^37–39^, the selected noise added to this data. In this scenario Gaussian noise is selected as first layer of encryption.

**Fig. 7:**
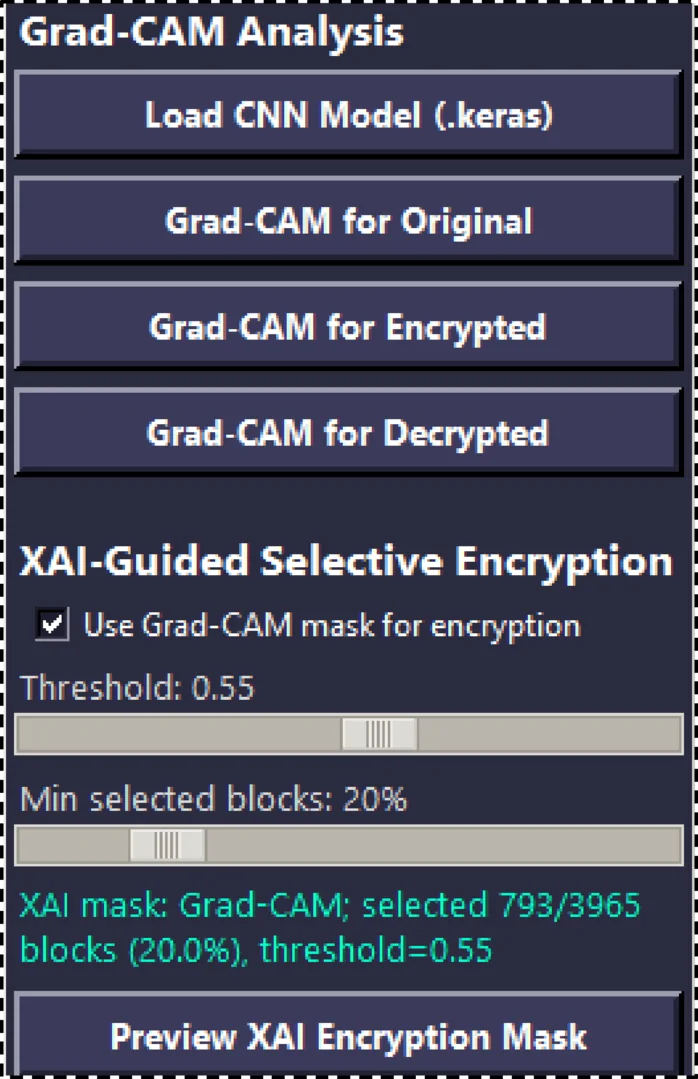
XAI and Grad-CAM analysis buttons

The proposed convolutional neural network (CNN) model in this research work was trained using the constructed dataset. The training process involved random splitting of the data into training and testing sets in an 80/20 proportion while maintaining a stratified splitting strategy to ensure balanced class distribution. In this study, the proposed model was designed in the structure shown in the Fig. 7 that included convolution layers for feature extraction, pooling layers to reduce dimensions and prevent overfitting, and fully connected layers for classification. To train the network, we used the Adam optimization algorithm, the Sparse Categorical Cross entropy loss function, the batch size of 8, and 30 epochs, while the early stopping mechanism was applied to monitor the validation accuracy and prevent overfitting. As data augmentation, rotations, horizontal flipping, cropping, and brightness adjustments were used. The trained dataset contains 2768 sample, divided to 2214 trained sample and554 test sample.

However, even after the decoding process, the image retained those features that distinguished it from the original image particularly in the middle part of the chest. In addition, there was some alteration in the positions including an increased activity level in the top region and a reduction in the average activity from 0.064 to 0.032. Thus, the explanation of the image was almost the same as that of the original image explanation.

**Fig. 8:**
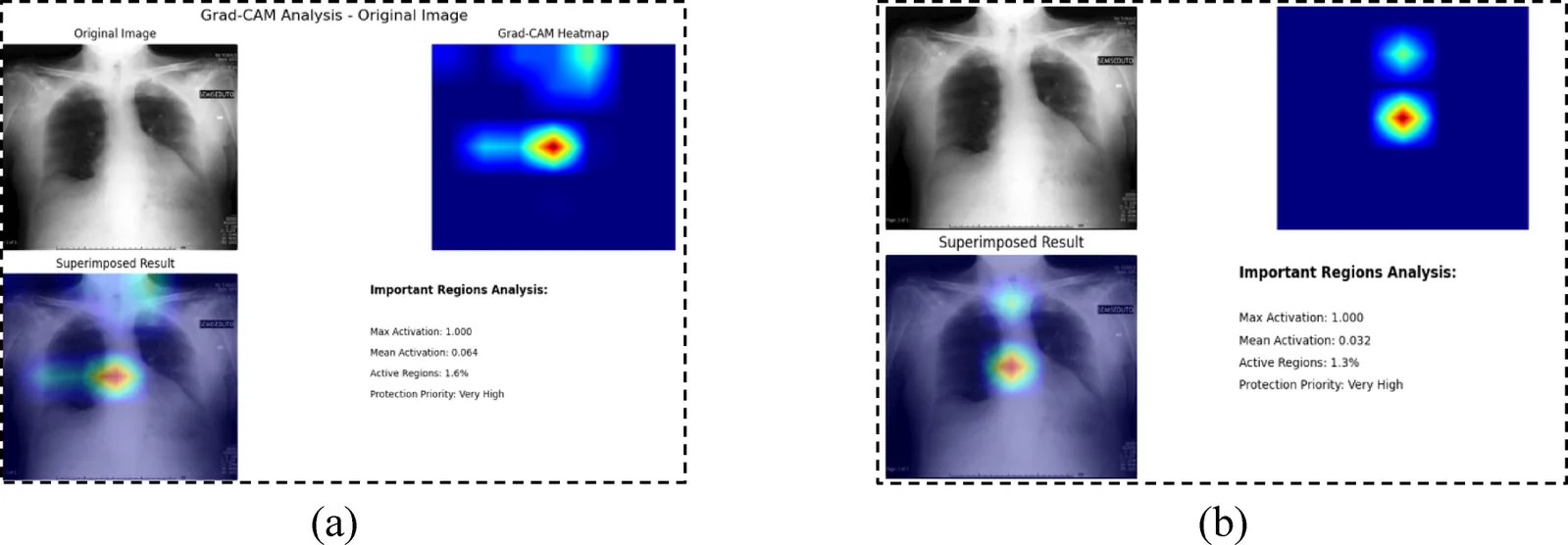
(**a**) Original image grad-CAM analysis. (**b**) Decrypted image grad-CAM analysis

The noise assigned at *σ =5* and noisy data is encrypted using the (8 × 8) blocks, at the receiving end the image was decrypted (Fig. 8a), the resulting satisfying PSNR is 34.24 dB, SSIM 0.8760, BER 0.2579% and the deformation factor is 0.1240. Finally use the suitable filter to extract the original image (Fig. 8b).

**Fig. 9:**
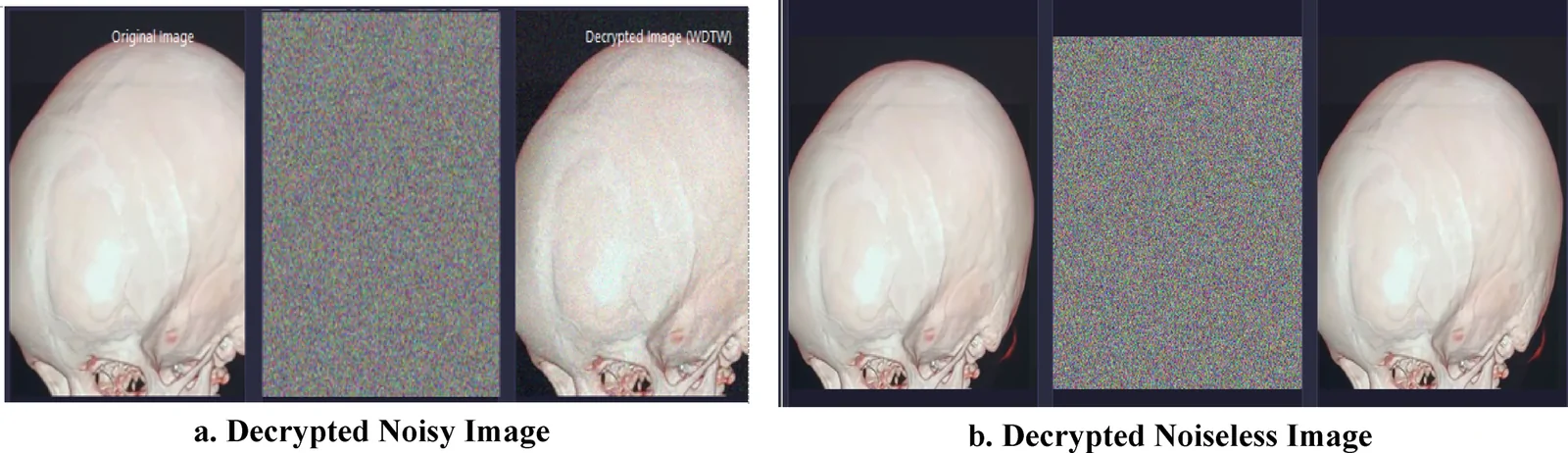
Decrypted image

According applying filter that in Fig. 9 results improvements in all parameters as show in listed Table 2 the PSNR is improved to 42.14 dB, SSIM is improved to 0.9807, BER is improved to 0.2042 and the deformation factor reduced 0.0193 as shown in quality metrics as show in Fig. 9.

**Table 2:** Comparison between noisy and noiseless received image.

	Noisy	Noiseless
PSNR	34.24 dB	42.14 dB
SSIM	0.8760	0.9807
BER	0.2579%	0.2042%
Deformation factor	0.1240	0.0193

**Fig. 10:**
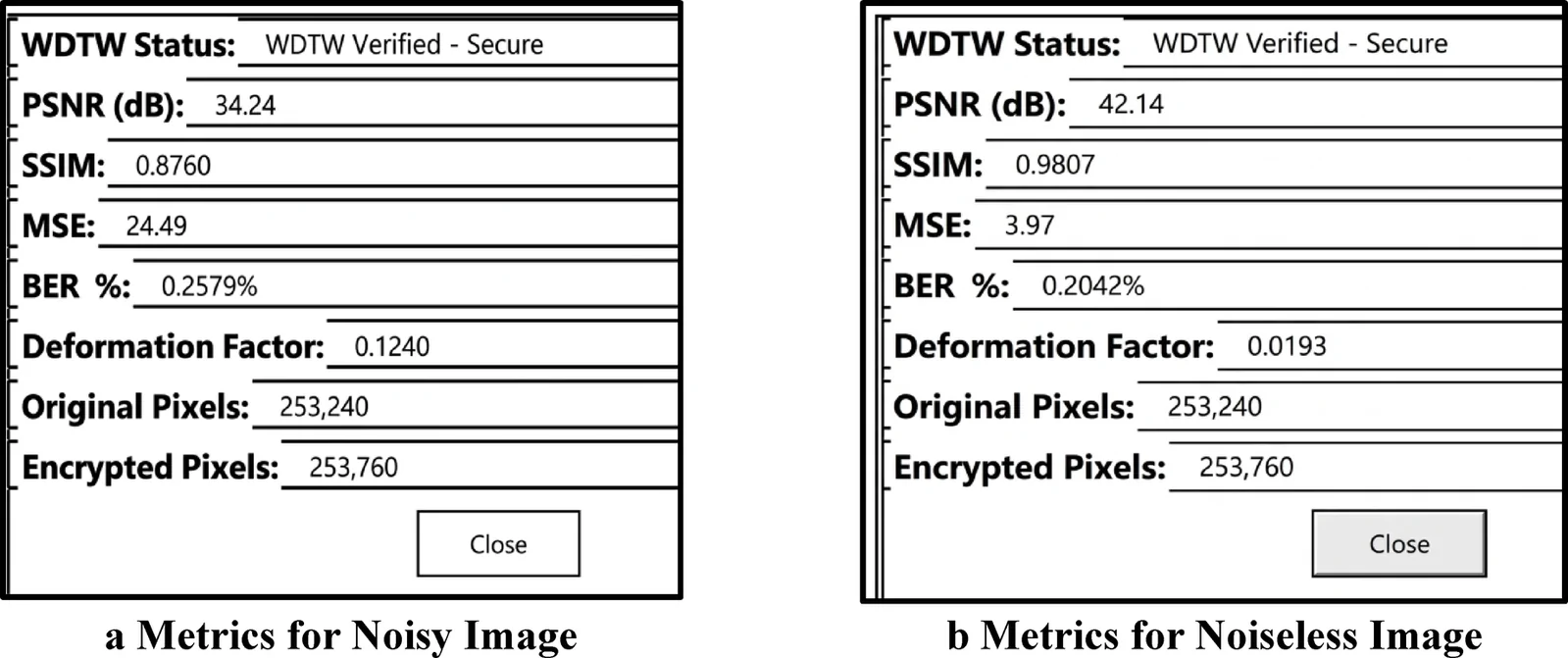
Quality metrics

The metrics window of Fig. 10, the first line shows the type of encryption method and the last two lines show number of original pixels and encrypted pixels. Number of encrypted pixels is greater than original pixels due to the padding pixels that added through the encryption.

### The system validation scenario

**Fig. 11:**
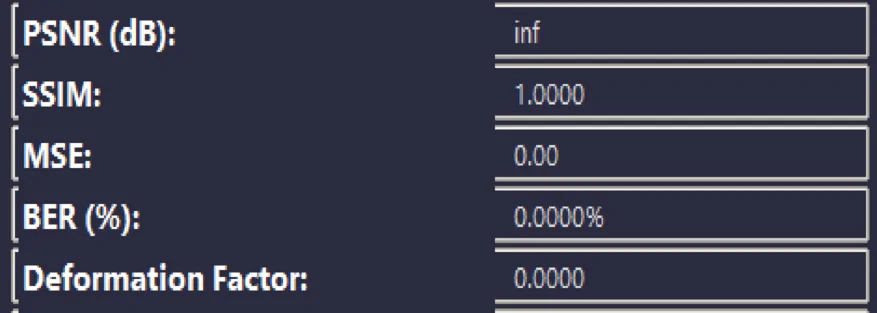
System validity

For testing the proposed algorithm, measures the metrics for decrypted image without adding noise through transmission as show Fig. 11, the result shows that all parameters achieve ideal values. The system parameters matrices (PSNR, SSIM, BER and Deformation factor) are used to display the proposed system features.

**Fig. 12:**
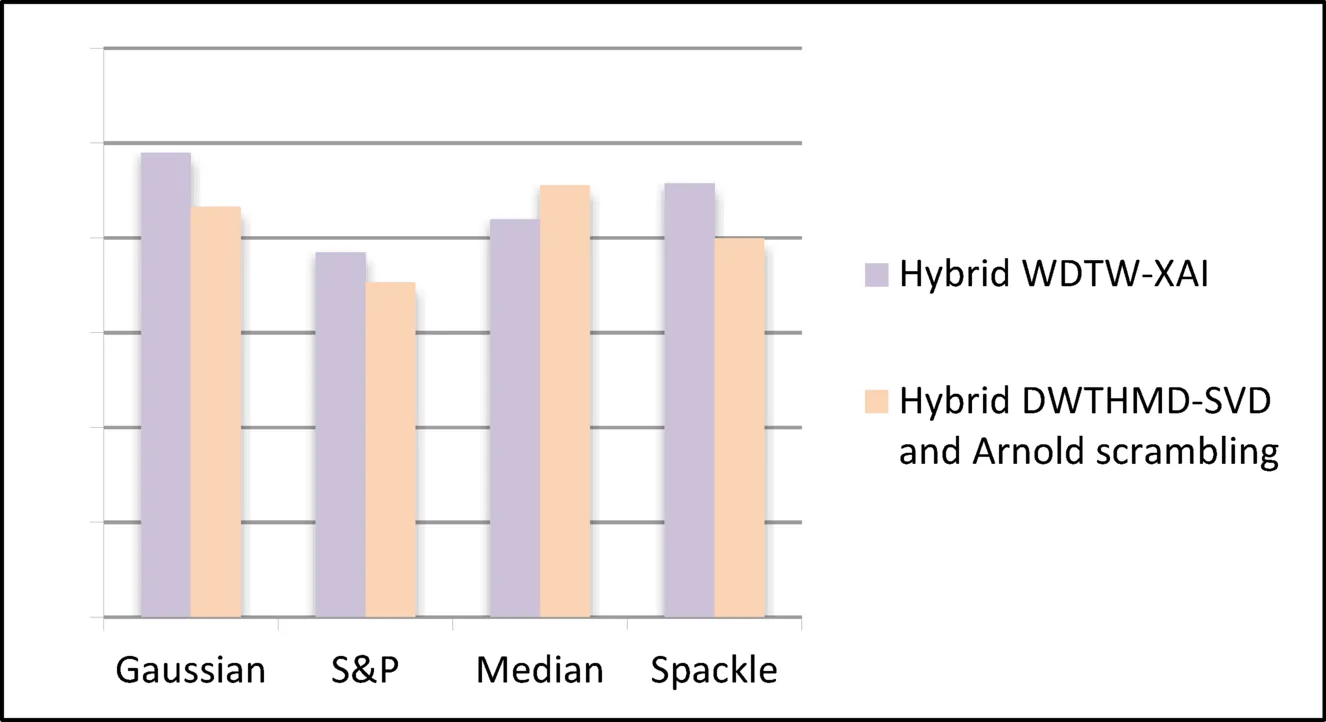
PSNR comparison between Hybrid XAI-WDT and hybrid DWTHMD-SVD and Arnold scrambling

**Fig. 13:**
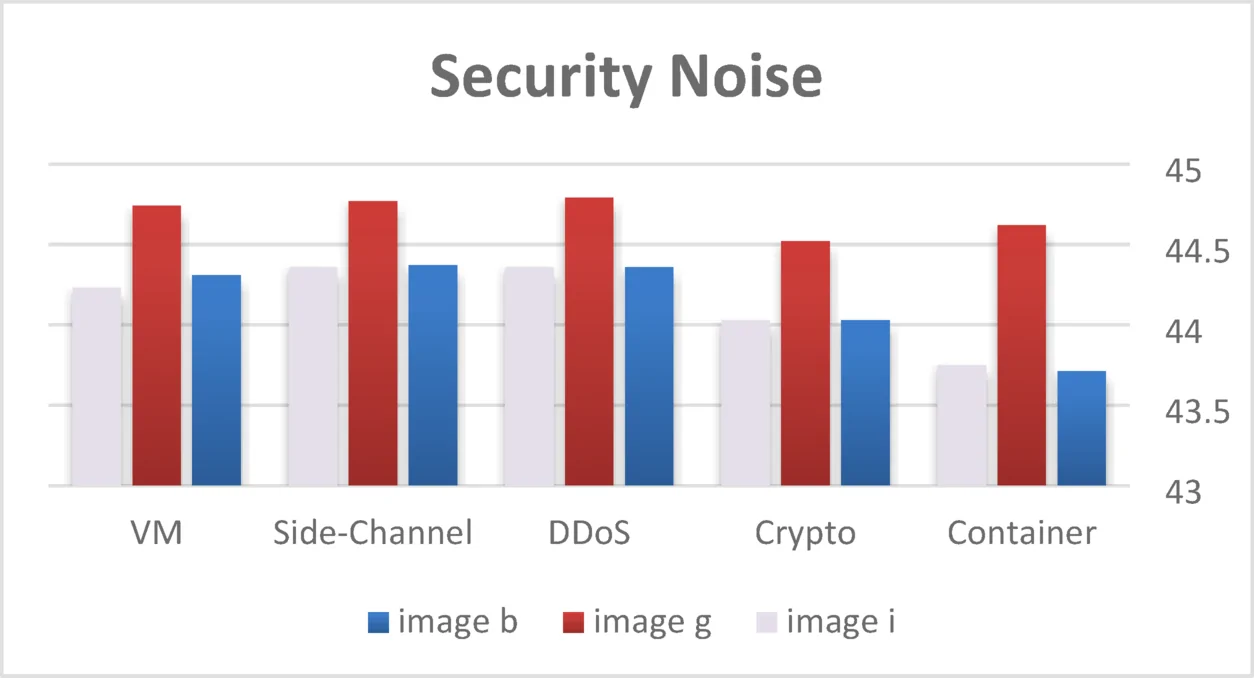
Security noise PSNR

The proposed algorithm compared with hybrid DWTHMD-SVD and Arnold scrambling at different types of noise, the results show that the proposed algorithm Hybrid XAI-WDTW achieves PSNR better than the other algorithm (Figs. 12 and 13; Tables 3 and 4).

**Table 3:** PSNR of proposed algorithm vs. hybrid DWTHMD-SVD and Arnold scrambling.

	Hybrid WDTW-XAI (dB)	Hybrid DWTHMD-SVD and Arnold scrambling (dB)
Gaussian	48.47	36.38
S&P	33.14	33.32
Median	33.63	39.63
Spackle	45.11	41.33

**Table 4:** Security noise evaluation.

	Image b (dB)	Image g (dB)	Image i (dB)
Container	43.71	44.62	43.75
Crypto	44.03	44.52	44.03
DDoS	44.36	44.79	44.36
Side-Channel	44.37	44.77	44.36
VM	44.31	44.74	44.23

**Fig. 14:**
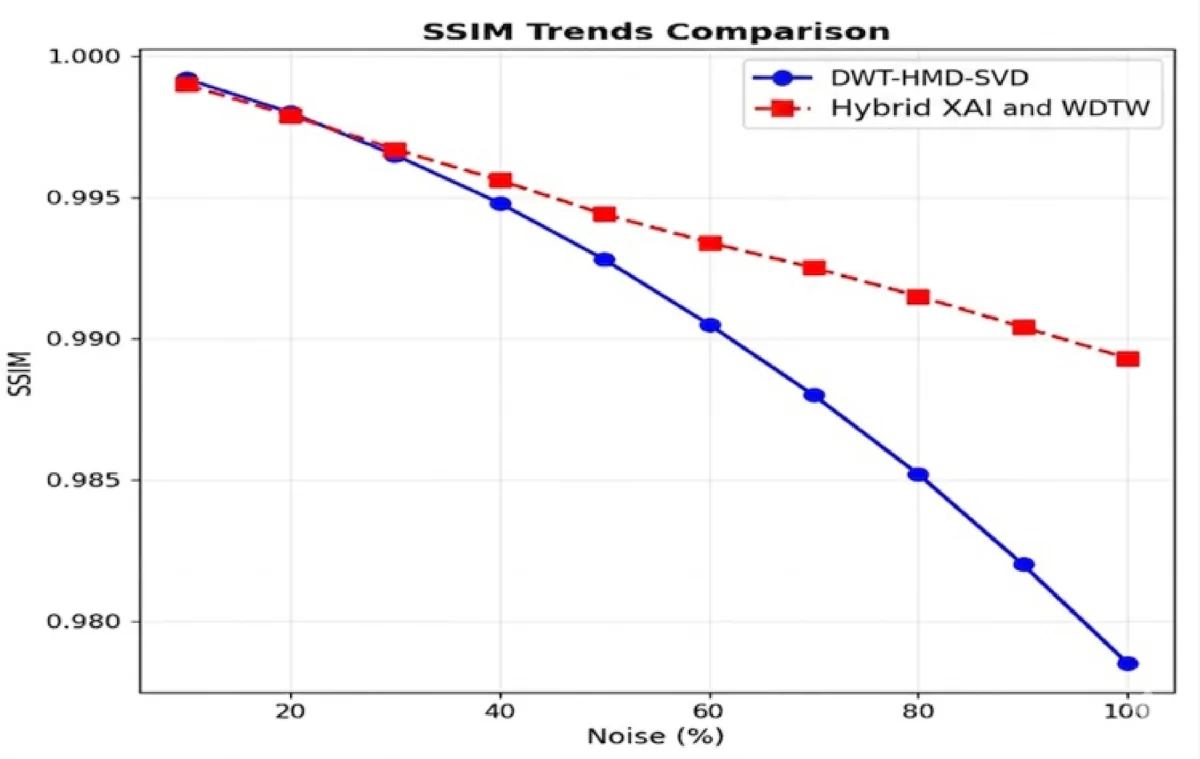
Similarity of decrypted image

Figure 14 shows that SSIM value gradually decreases with increasing noise levels in both methods, but the Hybrid XAI-WDTW approach maintains higher levels of structural similarity compared to the other method, especially at high noise levels. This suggests that the hybrid XAI-WDTW method is more noise-resistant and preserves image quality better.

**Fig. 15:**
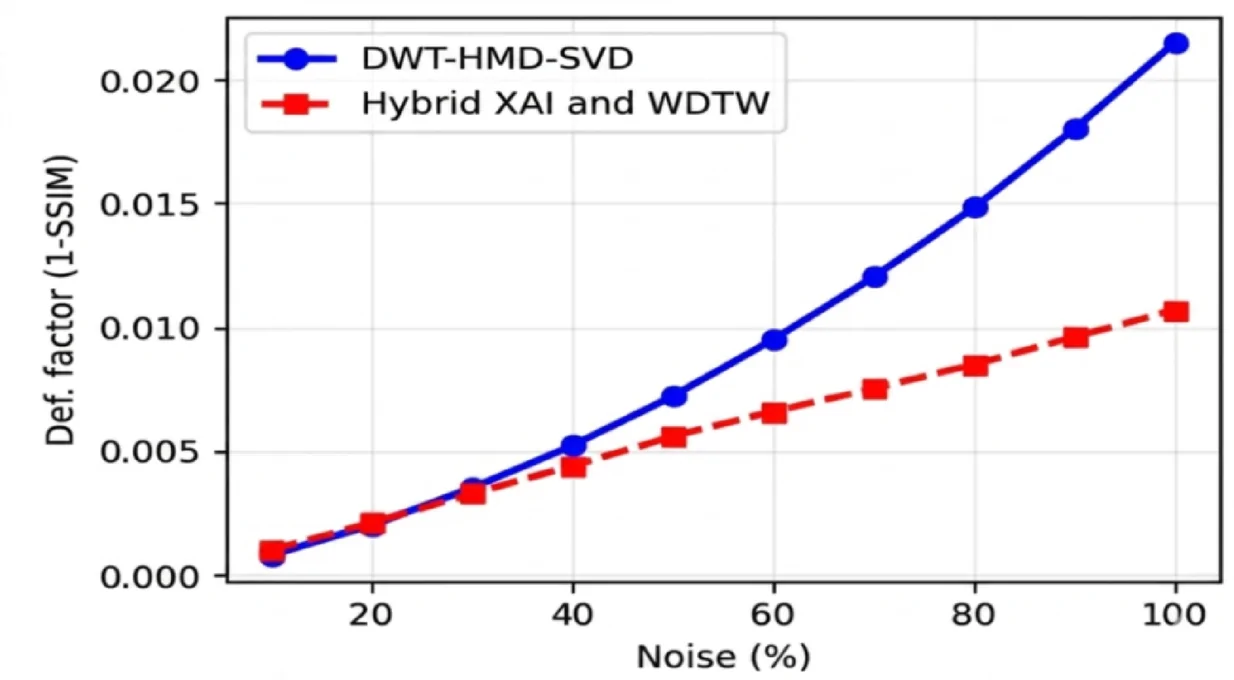
Performance for similarity

Where, Noise level represents sigma (*σ )* percentage, typically corresponding to the stander deviation of the noise distribution (Fig. 15).

In order to test the security strength of the suggested encryption algorithm based on wavelet transform technique (WDTW) in the cloud computing environment, the following analysis using standard tests was carried out for the original and encrypted images: entropy test, differential attack testing (NPCR/UACI), spatial correlation coefficient test, and key sensitivity test.

#### Entropy test

Entropy is the measure of randomness of pixels, and the greater the value (closer to 8 bits per pixel), the less predictable the data is and more resistant to brute force attacks. The analysis found that the entropy of the original image is 6.016028 bits, and it considerably increased up to 7.906129 bits in case of the encrypted image. Such an increase in the entropy indicates that the redistribution of pixels in case of the suggested WDTW algorithm is almost perfect and random.

#### Resistance against differential attack (NPCR & UACI)

The process of differential attack is one of the most dangerous attacks to any system of encryption, where the attacker tries to trace the influence of the change of one pixel of the initial image to its encrypted form. To protect the image from this type of attack, NPCR and UACI were calculated. It was found that the value of NPCR amounted to 99.6337%, which is higher than the critical value 99.60%. At the same time, the value of UACI equaled 33.1266%, being very close to the optimal value of 33.46%. Thus, it can be concluded that the algorithm is very sensitive to any change in the initial image.

#### Spatial correlation coefficient analysis

The main property of statistical attacks is the high degree of correlation between neighboring pixels in the image. Since natural images contain many correlated elements (the correlation coefficient is very close to + 1), to check how successful the encryption was in its ability to destroy such a correlation, correlation coefficients were calculated for both the initial and encrypted image along three axes: H (horizontal), V (vertical), and D (diagonal). For the original image, the correlation coefficients were high (H = + 0.991157, V = + 0.994132, D = + 0.986294). At the same time, for the encrypted image, the correlation coefficients dropped sharply to (H = 0.012931, V = − 0.006454, D = − 0.014912), almost equaling zero. Such a result means that the encryption algorithm successfully destroyed the image’s spatial structure so that an attack on the adjacent pixel correlation could not be carried out.

#### Key sensitivity analysis

In order for an encryption system to be regarded as safe, any minute changes made to the encryption key, including changes to a single bit, should produce a totally different output during the process of decryption. To conduct such a test, the image was first decrypted using the key that is exactly the same as the initial key and then decrypted using another key which differs from the former by only one bit. NPCR, and UACI values were calculated between the two decrypted images. It turns out that NPCR value reaches 99.6126% and UACI reaches 33.5066%. These values are quite similar to those that we got when compared the original image to the encrypted one. This proves the extremely high sensitivity of WDTW system to the key, since even the slightest difference in the key makes it impossible to decrypt the image.

**Fig. 16:**
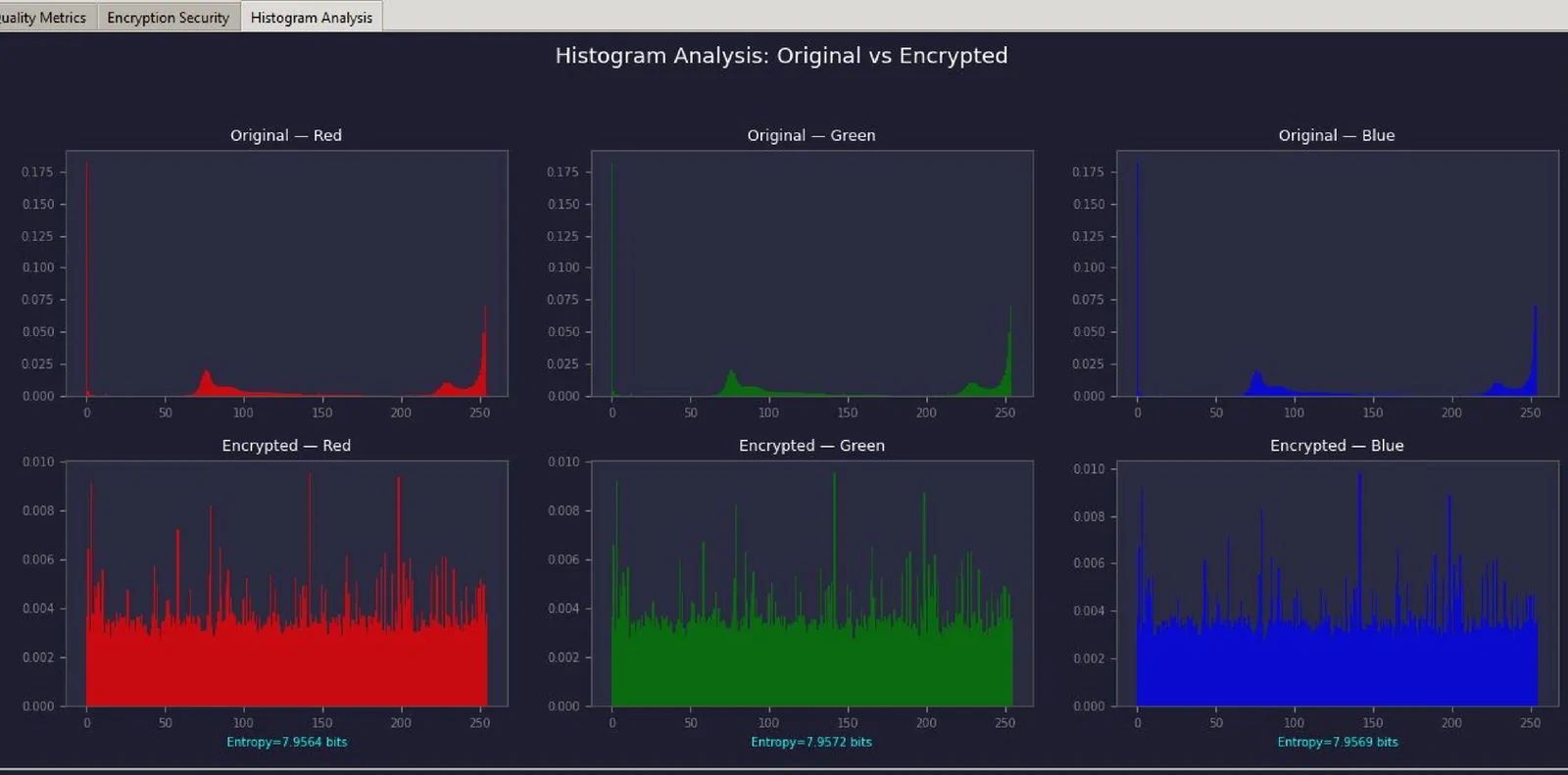
Histogram analysis of original and encrypted

Figure 16 shows the histogram analysis for the RGB channels of both the original and the encrypted images. It demonstrates the efficiency of the encryption algorithm. Histograms of the original images have very concentrated distribution with sharp peaks at certain levels of intensity in the lower range of 0–50 and higher range of 200–255. It is the distinguishing property of natural images and it shows that the entropy of the image is rather low. In contrary, the histograms of the encrypted images show drastic change to the more or less uniform distribution through the whole range of the intensities from 0 to 255 with very flat peaks and increased stochasticity. The entropy of the encrypted image channels (Red: 7.9564 bits, Green: 7.9572 bits, Blue: 7.9569 bits) is close to the maximum possible value of 8 bits with deviation of just about 0.5% from it. The increase in the entropy of approximately 1.9 bits in comparison with the original image is the proof of almost complete randomization of the pixels intensities.

**Fig. 17:**
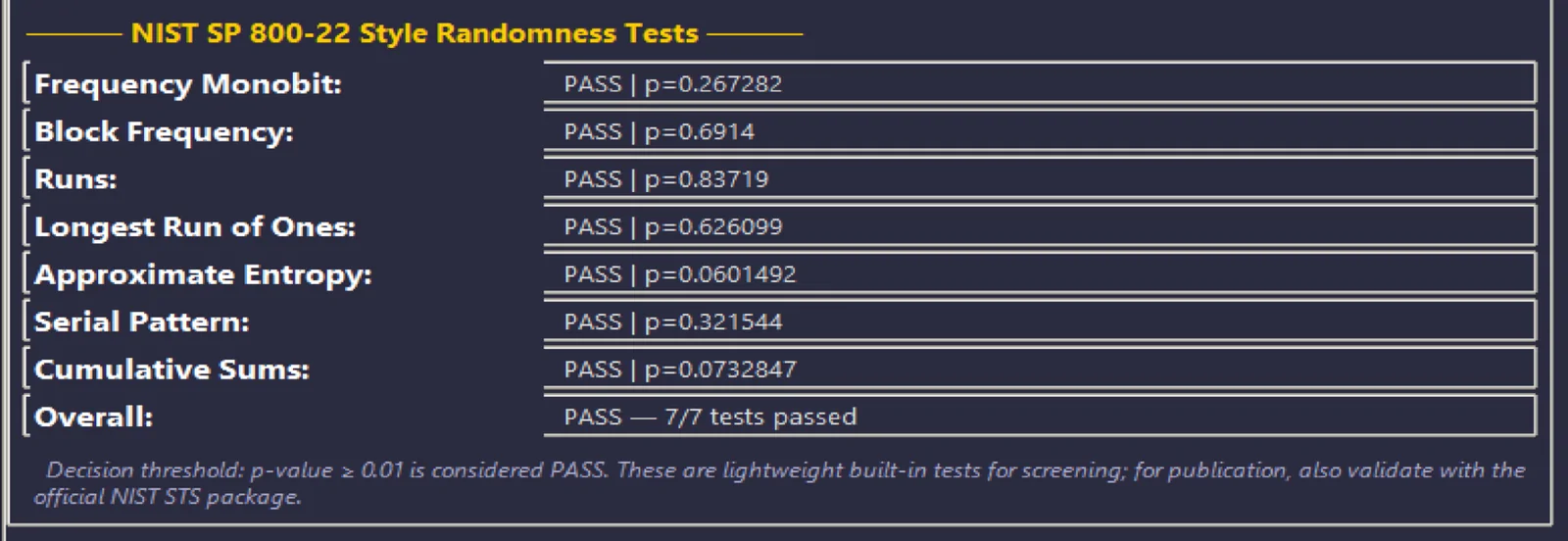
NIST randomness tests evaluation

The encryption process passed successfully seven randomness test methods suggested by NIST SP 800-22 in terms of *p* value higher than the chosen significance level of 0.01. This means that there is no statistical deviation from randomness detected for the studied features such as frequency, runs, block frequency, longest-run, approximate entropy, serial and cumulative sums (Fig. 17).

**Fig. 18:**
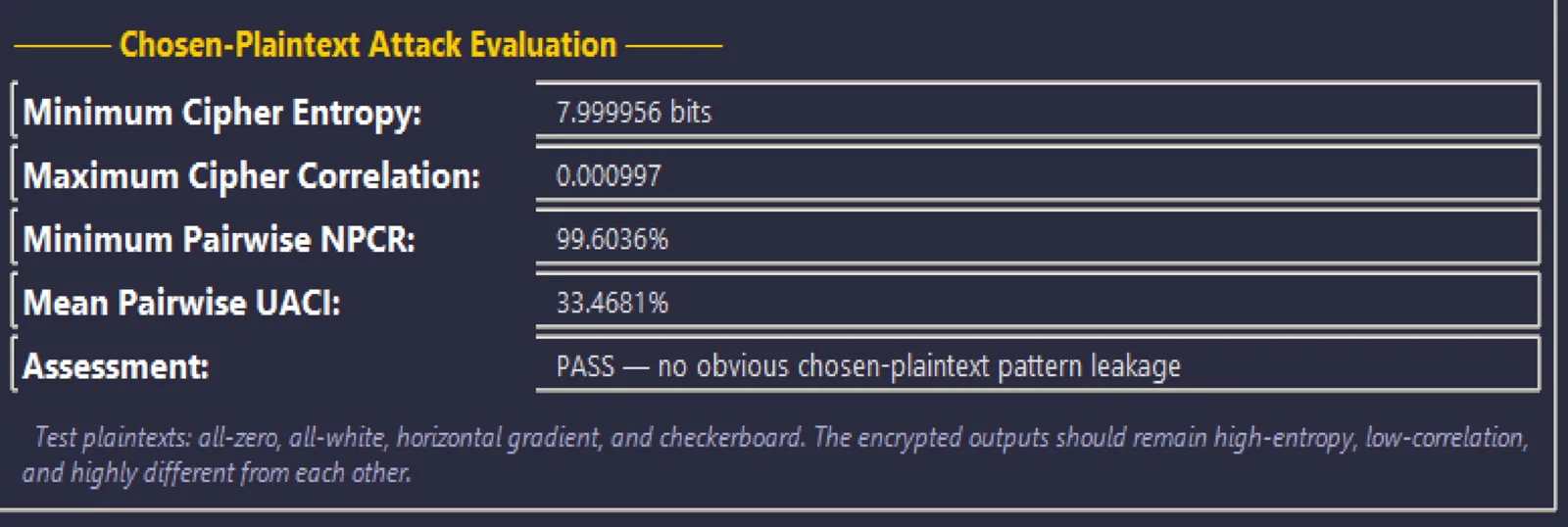
Chosen-plaintext attack evaluation

The chosen-plaintext evaluation showed strong resistance to structured input patterns. Across the all-zero, all-white, gradient, and checkerboard plaintexts, the minimum ciphertext entropy reached 7.999956 bits, while the maximum adjacent-pixel correlation remained as low as 0.000997. In addition, the minimum pairwise NPCR was 99.6036%, and the mean pairwise UACI was 33.4681%, indicating that different structured plaintexts produced highly dissimilar ciphertexts with near-random statistical characteristics. These findings suggest no obvious chosen-plaintext pattern leakage under the tested conditions (Fig. 18).

Table 5 show comparison between hybrid WDTW-XAI and hybrid DWTHMD-SVD and Arnold scrambling algorithm at the same dataset. The result shows the Table 6.

**Table 5:** Quantitative comparison of proposed model vs. other methods.

Refs.	Entropy	NPCR	UACI	PSNR	SSIM	Correlation coefficient
5	–	73.66%	77%	38.38 dB	0.76	–
6	–	–	–	58.27 dB	0.942	0.90
24	7.87	99.592%	34.268%	–	–	–
Proposed	7.906129	99.6337%	39.1643%	48.47 dB	0.9914	0.99

**Table 6:** Comparison between proposed algorithm and other algorithm.

	Hybrid DWT-HMD-SVD and Arnold scrambling^7^	Hybrid WDRW-XAI	Reference range
PSNR	38.38 dB	48.47 dB	Typical values: 30–50 dB^25^
SSIM	0.76	0.9914	0–1 (close to 1 is the best)
BER	–	0.0313%	100–0% (less is the best)
Deformation factor	0.24	0.0086	0–1 (less is the best)

## Conclusion

In this research, a novel architecture for a robust image processing, including encryption, noise addition, auto-adaptive filtering, and visual interpretation, was developed. The offered encryption scheme consists of WDTW encryption and block-wise scrambling, which allows for encrypting the data and adding different types of noise, including Gaussian, salt-and-pepper, spackle and median noise. The “Auto” mode of the proposed tool automatically selects the optimal filtering algorithm based on the characteristics of the noise. The simulated results obtained in the course of evaluating the performance of the offered method based on PSNR, SSIM, deformation factor and BER metrics showed that the auto-adaptive filtering approach works efficiently for recovering the initial image under different noise levels, delivering high PSNR scores and preserving perceptual structures. The implementation of the Grad-CAM model allows the user to receive clear visual information about the areas of interest. A GUI facilitates a seamless user experience by providing easy interaction and exporting of data in an appropriate format. The secure cloud image processing system incorporating Hybrid WDTW- XAI to detect the critical areas in the image has been successfully achieved. We observe that the SSIM metric maintains a nearly constant value close to 1, indicating that structural similarity remains high despite increasing noise. The PSNR metric gradually decreases as noise levels rise, reflecting a decline in image quality in terms of signal-to-noise ratio. The BER and Deformation Factor metrics increase almost linearly with increasing noise, which is expected since they measure the amount of error or distortion. Overall, the graph demonstrates an inverse relationship between image quality and noise. The simulation results have been shown the proposed system provides better data recovery than hybrid DWTHMD-SVD and Arnold scrambling. The PSNR of 26.26%, SSIM of 23.7% and 23.7% of deformation factor, also achieves the best BER the results are in satisfactory agreement with the selected dataset. A one-bit modification of the master key produced an NPCR of 99.6126%, and a UACI of 33.5066%, demonstrating strong key sensitivity. In the controlled one-bit plaintext differential test, the corresponding NPCR, and UACI values were 99.8088%, and 33.5244%, showing that a minimal plaintext change caused an approximately random change in the cipher text. The encrypted bit stream also passed all seven implemented NIST SP 800-22-style screening tests, including frequency, block frequency, runs, longest run of ones, approximate entropy, serial pattern, and cumulative sums, with all *p* values remaining above 0.01. In addition, the chosen-plaintext evaluation using all-zero, all-white, gradient, and checkerboard images produced a minimum cipher text entropy of 7.999750 bits, a maximum absolute correlation of 0.001754, a minimum pairwise NPCR of 99.6020%, and a mean pairwise UACI of 33.4857%. These findings indicate that the encrypted outputs retained high entropy, low spatial correlation, strong plaintext and key sensitivity, and no obvious statistical leakage for the tested structured plaintexts. Exact reversibility was also verified, with HMAC validation passing.

## Data Availability

The datasets generated and/or analysed during the current study are available in the^25–27^ repository, https://www.kaggle.com/datasets/pranavraikokte/covid19-image-dataset , https://www.kaggle.com/datasets/maedemaftouni/covid19-ct-scan-lesion-segmentation-dataset—The datasets used and/or analysed^28^ during the current study available from the corresponding author on reasonable request.
